# Plexin-B2 facilitates glioblastoma infiltration by modulating cell biomechanics

**DOI:** 10.1038/s42003-021-01667-4

**Published:** 2021-01-29

**Authors:** Yong Huang, Rut Tejero, Vivian K. Lee, Concetta Brusco, Theodore Hannah, Taylor B. Bertucci, Chrystian Junqueira Alves, Igor Katsyv, Michael Kluge, Ramsey Foty, Bin Zhang, Caroline C. Friedel, Guohao Dai, Hongyan Zou, Roland H. Friedel

**Affiliations:** 1 Friedman Brain Institute, Nash Family Department of Neuroscience, Icahn School of Medicine at Mount Sinai, New York, NY USA; 2 Department of Bioengineering, Northeastern University, Boston, MA USA; 3 Department of Genetics and Genomic Sciences, Icahn School of Medicine at Mount Sinai, New York, NY USA; 4 Institut für Informatik, Ludwig-Maximilians-Universität München, Munich, Germany; 5 Department of Surgery, Robert Wood Johnson Medical School, Rutgers University, New Brunswick, NJ USA; 6 Department of Neurosurgery, Icahn School of Medicine at Mount Sinai, New York, NY USA

**Keywords:** Cancer microenvironment, Cellular motility, CNS cancer

## Abstract

Infiltrative growth is a major cause of high lethality of malignant brain tumors such as glioblastoma (GBM). We show here that GBM cells upregulate guidance receptor Plexin-B2 to gain invasiveness. Deletion of Plexin-B2 in GBM stem cells limited tumor spread and shifted invasion paths from axon fiber tracts to perivascular routes. On a cellular level, Plexin-B2 adjusts cell adhesiveness, migratory responses to different matrix stiffness, and actomyosin dynamics, thus empowering GBM cells to leave stiff tumor bulk and infiltrate softer brain parenchyma. Correspondingly, gene signatures affected by Plexin-B2 were associated with locomotor regulation, matrix interactions, and cellular biomechanics. On a molecular level, the intracellular Ras-GAP domain contributed to Plexin-B2 function, while the signaling relationship with downstream effectors Rap1/2 appeared variable between GBM stem cell lines, reflecting intertumoral heterogeneity. Our studies establish Plexin-B2 as a modulator of cell biomechanics that is usurped by GBM cells to gain invasiveness.

## Abstract (web summary)

Huang et al demonstrate that glioblastoma cells upregulate axon guidance molecule Plexin-B2 to gain invasiveness and that Plexin-B2 promotes glioblastoma cell infiltration along axon fiber tracts in intracranial transplant models by modulating cellular biomechanics.

## Introduction

Glioblastoma (GBM), the most common malignant brain tumor in adults, is highly lethal due to its infiltrative growth behavior, which prevents complete surgical resection^1,2^. To initiate invasion, GBM cells must loosen attachment to neighboring cells and matrix; they then face the challenge of negotiating through tight interstitial spaces while maintaining traction on the substrate surface. The cellular and molecular mechanisms of how invading GBM cells achieve the necessary mechanocompliance remain unclear. GBM cells are known to invade along microvasculature and axon fiber tracts^3,4^, but the molecular factors influencing the choice of preferred migratory paths are poorly understood. Such understanding will be fundamental for identifying molecular targets to curb GBM dissemination.

Aside from biochemical cues such as chemokines, cell migratory behavior is also governed by the physical characteristics of the surrounding environment. Interestingly, when faced with different substrate rigidity, most cell types migrate towards higher substrate stiffness, a behavior known as durotaxis^5,6^. During GBM progression, as the tumor microenvironment gradually stiffens^7^, GBM cells must attenuate their durotactic bias in order to dissociate from stiff tumor bulk and invade softer brain parenchyma. Axon guidance molecules play a central role in mediating neural migration during brain development^8^. As tumor cells frequently usurp developmental pathways to fuel their expansion, we set out to investigate how guidance receptors may contribute to GBM invasiveness.

Plexins are axon guidance receptors that bind to semaphorin ligands and regulate diverse cellular interactions in development and adult physiology^9–11^. Plexins signal through small G proteins of the Rap and R-Ras families, which are pleiotropic regulators of cell adhesion and cytoskeletal dynamics^12–15^. Aberrant expression of plexins and semaphorins has been observed in multiple types of cancer and is associated with tumor malignancy^16,17^. Plexin-B2 is a particularly strong candidate as a promoter of GBM invasion, as it was originally discovered due to its upregulation in brain cancers^18^, and subsequent analysis confirmed Plexin-B2 as a biomarker for malignant glioma based on patient data^19^. Fitting with a role in GBM invasion is also the fact that Plexin-B2 exerts a critical function in regulating neuroprecursor migration during brain development^20,21^. Our own studies have also revealed a link of high expression of Plexin-B2 with poor survival of GBM patients and that Plexin-B2 enhances the migratory capacity of GBM cells^22^. However, these earlier studies were mainly conducted in traditional GBM cell lines cultured in serum-containing medium, i.e., U87MG and LN229, which do not display infiltrative growth in intracranial transplants, and are thus not well-suited to study GBM invasiveness. Moreover, the underlying mechanisms of how Plexin-B2 promotes GBM invasion are not understood.

Interestingly, Plexin-B2 has also been shown to function as an entry receptor for angiogenin, a secreted RNA-binding protein with multiple tumorigenic effects. However, binding and internalization of angiogenin via Plexin-B2 do not appear to activate Plexin-B2 and its downstrem signaling components as the canonical semaphorin ligands do^23^.

Here, we investigated how Plexin-B2 enables GBM cells to gain invasiveness by utilizing a set of patient-derived GBM stem cell (GSC) lines, which preserve major pathophysiology of parental GBM, including infiltrative invasion in vivo^24–27^. We demonstrate in intracranial transplant models that fine-tuned Plexin-B2 activity is required for diffuse infiltration of GBM cells along axon fiber tracts. Remarkably, Plexin-B2 ablation not only limits GBM spread but also alters invasion patterns and migratory paths. Through a series of mechanosensitive assays as well as molecular studies, we show that GBM cells gain invasiveness by usurping Plexin-B2 signaling to adjust cell biomechanics.

## Results

### Diffuse infiltration of GBM cells along axon fiber tracts in intracranial transplan**t**s

To study the mechanisms of diffuse invasion of GBM cells, we utilized a set of patient-derived glioma stem cell lines (GSCs), SD1–SD4, which had been established and maintained in neural stem cell media^22^. The molecular diversity of these GSCs reflects high intertumoral heterogeneity of GBM: for instance, western blotting (WB) revealed distinct expression profiles of receptor tyrosine kinases–EGFR in SD1, EGFR and MET in SD2, and PDGFRα in SD3 and SD4, and RNA-sequencing (RNA-seq) analysis indicated three different transcriptional subtypes^28^ (Supplementary Fig. 1a, b). All GSC lines are IDH1/2 wild type (Supplementary Fig. 1b), which is typical for adult primary GBM^29^.

**Fig. 1:**
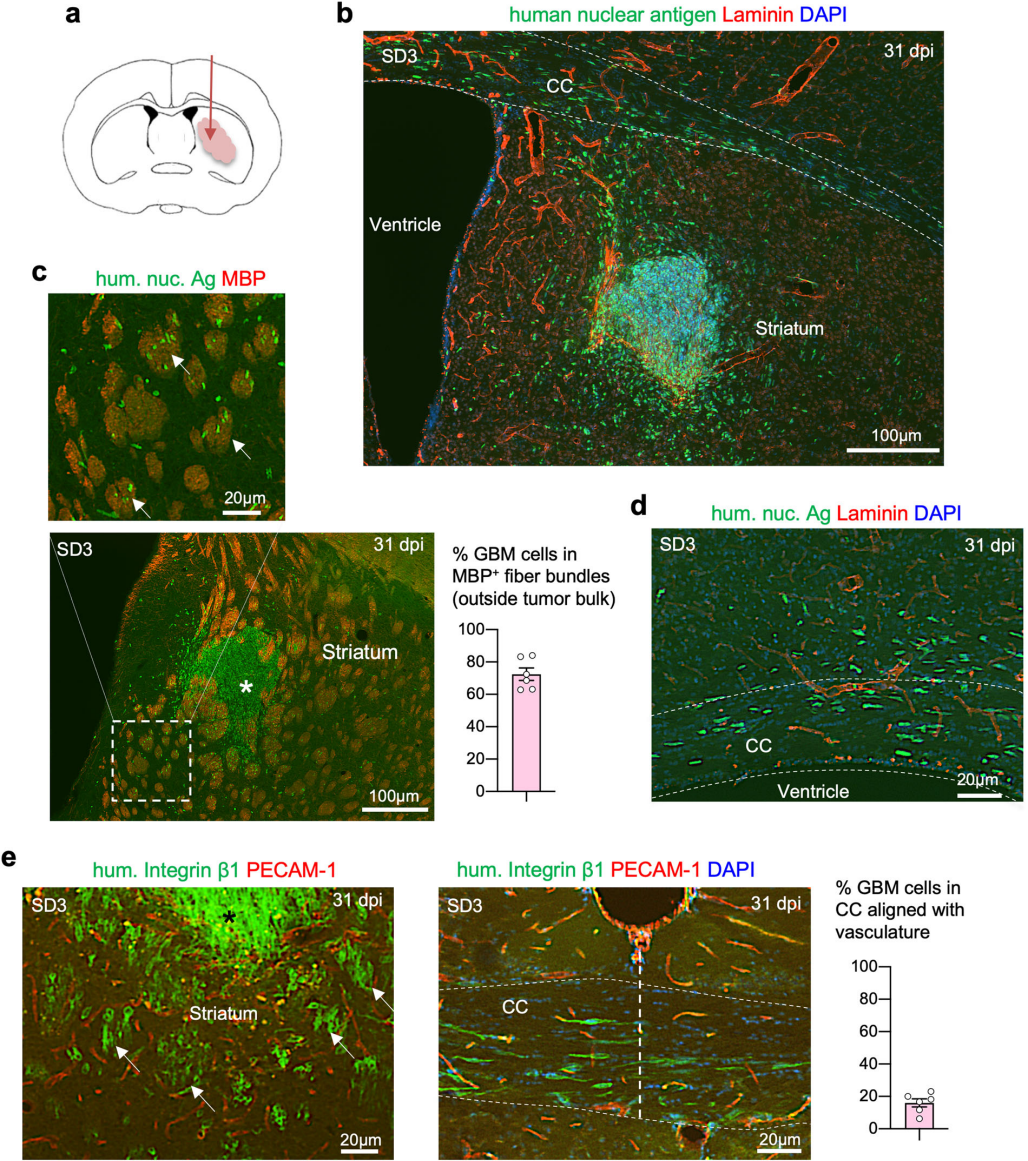
Diffuse GBM infiltration along axon fiber tracts in intracranial GSC transplants. **a** Diagram of orthotopic transplantation of patient-derived GSCs into the striatum of SCID mouse host. All images in this figure panel are from GSC line SD3 at 31 days post implantation (dpi). **b** Immunofluorescence (IF) image of coronal brain section with SD3 GSC transplant at 31 dpi, showing diffuse infiltration of tumor cells expressing human nuclear antigen (hum. nuc. Ag) in the striatum and along corpus callosum (CC, outlined by dashed lines). Laminin IF highlights the basal lamina ensheathing blood vessels. **c** Enlarged IF image shows a clear preference of invading GBM cells (hum. nuc. Ag^+^) for striatal axon fiber bundles (arrows), highlighted by IF for myelin basic protein (MBP). Numerous individual tumor cells had migrated away from tumor bulk (asterisk). Quantification indicates that in areas outside tumor bulk, greater than 70% of infiltrating cells are found in MBP^+^ fiber bundles (*n* = 6 fields from three animals; mean ± SEM). **d** IF image of the ipsilateral area of SD3 GSC transplant shows GBM cells invading along fiber tracks of the corpus callosum (CC). Note the orientation of elongated nuclei of the invading GBM cells aligned with axon fiber trajectory, but not with blood vessel axis (laminin^+^). **e** IF images of ipsilateral striatum (left) and CC at the midline (right) from brain transplanted with SD3 GSCs. Tumor cells stained for human integrin β1, which highlights cell surfaces, show diffuse dissemination within striatal axon fiber bundles (arrows) surrounded at the periphery by microvasculature (PECAM-1^+^). Note that the orientation of striatal fiber bundles and adherent GBM cells appeared perpendicular to the coronal plane. Also note the fusiform shape of the invading GBM cells in the CC, with cell axes aligned with axon trajectory, but not with microvasculature. Quantification on the right indicates the percentage of cells in the corpus callosum that are aligned with vessels (*n* = 6 fields from three animals; mean ± SEM).

Upon transplantation into the striatum of immunocompromised mice, all four GSC lines generated aggressive GBM, although their expansion rates appeared different. For instance, while SD2 GSCs gave rise to a small tumor mass at 141 days post implantation (dpi), the bulk tumor mass formed by SD3 GSCs was already sizable by 31 dpi (Fig. 1a, b and Supplementary Fig. 1c). A hallmark shared by all GSC lines was their diffuse infiltration into host brains. Immunofluorescence (IF) staining for human nuclear antigen highlighted the wide dissemination of GBM cells not only in the striatum but also deep into the contralateral hemisphere along the corpus callosum (Fig. 1c, d and Supplementary Fig. 1c–e).

As GBM is known for perivascular invasion^30^, we examined the spatial relationship of invading tumor cells with blood vessels, which were visualized by IF for endothelial marker PECAM-1, or laminin, a matrix protein of the vascular basement membrane^31^. Unexpectedly, we observed no dominant vascular association of the invading GBM cells in the striatum or in the corpus callosum; instead, they exhibited a preference for axon fiber tracts, which was confirmed by IF for myelin basic protein (Fig. 1c–e). Within the striatum where neuronal tissue is interspersed with axon fiber bundles, GBM cells mainly resided inside the axon fiber bundles (Fig. 1e).

Consistently, in the corpus callosum, the nuclei of the invading GBM cells assumed an elongated morphology aligned with the axon fiber trajectory (Fig. 1d and Supplementary Fig. 1c–e). Cell surface staining by IF for human integrin β1 further outlined the fusiform shape of the migrating GBM cells in the same orientation as the axon fiber tracts but not as the blood vessel axes in the corpus callosum (Fig. 1e). Likewise, in the striatum, the nuclei of migrating GBM cells were largely aligned with axon fiber bundles which run perpendicular to the coronal plane (Fig. 1e). Staining with an isotype control antibody confirmed the specificity of IF for human integrin β1 (Supplementary Fig. 2). Taken together, in orthotopic GSC transplants, invading GBM cells disseminate widely and negotiate through tight interstitial spaces, taking up fusiform morphology and adhering to preferred migratory paths along axon fiber tracts.

### Plexin-B2 and semaphorin 4 expression in GSCs

**Fig. 2:**
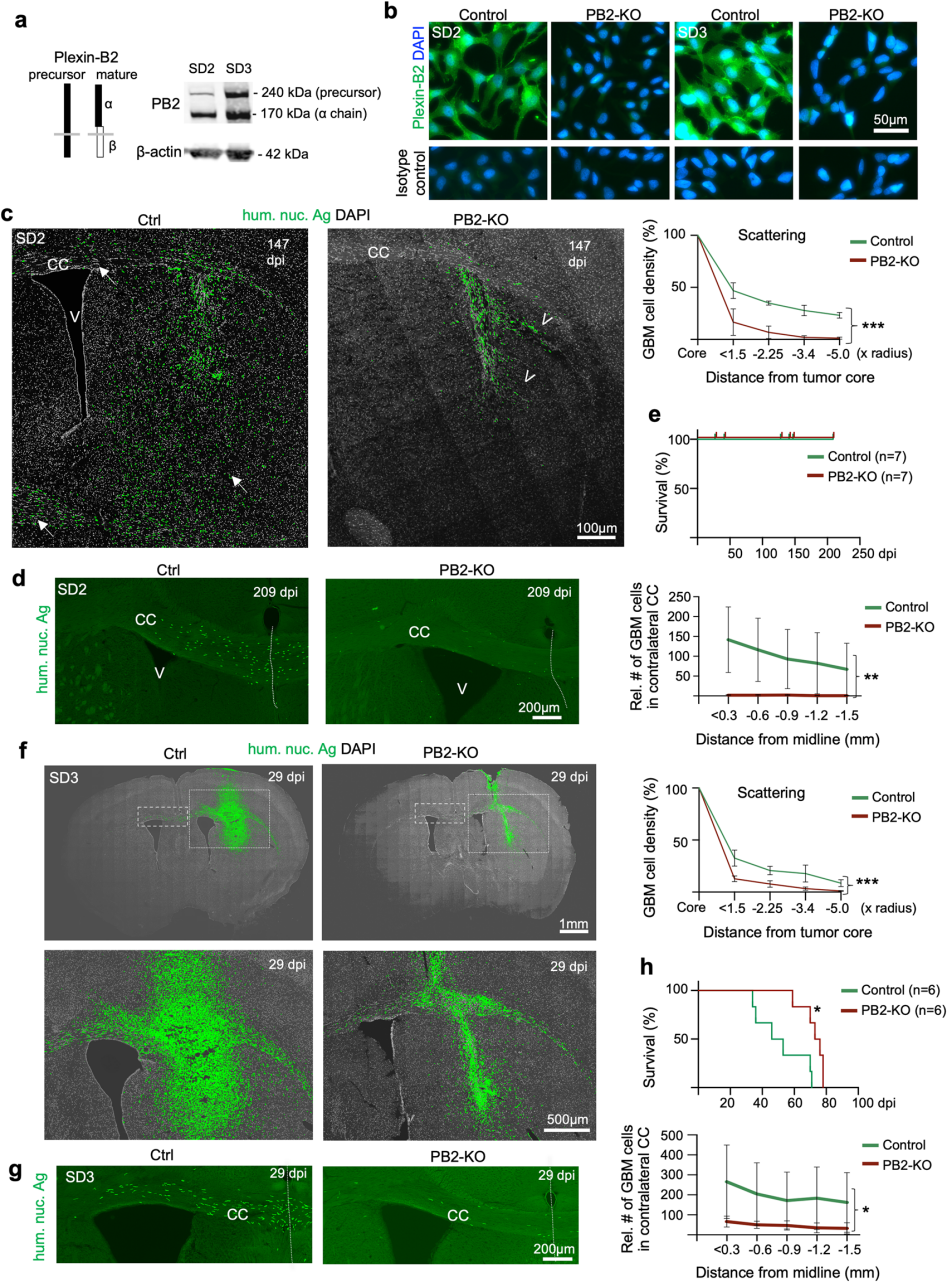
Plexin-B2 ablation limits GBM spread. **a** Diagram illustrating structure of Plexin-B2 precursor and mature form (during maturation, Plexin-B2 is cleaved into a non-covalently linked complex of α and β chains). WB with an antibody against the extracellular domain of Plexin-B2 shows a robust expression of Plexin-B2 in SD2 and SD3 GSCs. Note that cells typically express both precursor and mature forms of Plexin-B2, hence the double band pattern. **b** IF images of cultured GSCs demonstrate the absence of surface expression of Plexin-B2 (PB2) in cells with CRISPR KO. IF images of cells stained with isotype IgG control are shown in the bottom panels. **c** IF images of coronal brain sections with SD2 GSC transplants at 147 days post injection (dpi). Note the diffuse infiltration of tumor cells (hum. nuc. Ag^+^) in striatum and corpus callosum (CC) (arrows) in the control transplant, while PB2-KO GBM cells were mainly confined near the injection site. Also note tumor cell aggregation in collective migration streams at the tumor periphery in PB2-KO transplant (arrowheads), in contrast to the diffuse invasion pattern in control transplant. Quantifications on the right show the relative density of GBM cells (normalized to tumor core) in rings of increasing radius from the tumor core. *n* = 3 mice per genotype. Two-way ANOVA, ***P < 0.001. **d** IF images of the CC region show abundant tumor cells (hum. nuc. Ag^+^) crossing midline (dotted line) in control transplant at 209 dpi, but much fewer detectable tumor cells in contralateral CC in PB2-KO transplant. Quantifications show the relative abundance of GBM cells found in segments of 0.3 mm increment in contralateral CC. *n* = 4 mice per genotype. Two-way ANOVA, **P < 0.01. **e** Kaplan–Meier survival curves of mice transplanted with control or PB2-KO SD2 GSCs. No mice died for up to 209 dpi for either cohort (*n* = 7 mice per cohort), reflecting slow tumor expansion of SD2 GSCs in vivo. **f** IF images of coronal brain sections from mice transplanted with SD3 GSCs at 29 dpi. GBM cells with PB2-KO were more restricted in their infiltration than control cells. Quantifications on the right show the relative density of GBM cells (normalized to tumor core) in rings of increasing radius from the tumor core. *n* = 3 mice per genotype. Two-way ANOVA, ***P < 0.001. **g** Enlarged images of contralateral CC are shown for SD3 transplant. Midline is denoted by a dotted line. Quantifications show the relative abundance of GBM cells found in segments of 0.3 mm increment in contralateral CC. *n* = 3 mice per genotype. Two-way ANOVA, *P < 0.05. **h** Kaplan–Meier survival analysis shows that mice bearing intracranial transplants of PB2-KO SD3 GSCs survived significantly longer than mice with transplants of control GSCs: *n* = 6 mice per cohort; median survival 74.5 days vs. 49.5 days; *P = 0.014, Log-rank test.

To understand the contribution of Plexin-B2 to GBM invasion, we first examined its expression in GSCs and found that all four GSC lines abundantly expressed Plexin-B2 (Fig. 2a and Supplementary Fig. 3a), in agreement with earlier reports on Plexin-B2 upregulation in gliomas^19,22^. Class 4 semaphorins are putative ligands for Plexin-Bs, and we detected SEMA4B and SEMA4C expression in cultured GSCs by WB, indicating the possibility of paracrine stimulation of Plexin-B2 (Supplementary Fig. 3b). Other class 4 semaphorins were also expressed in GSCs, as shown by RNA-seq analysis (Supplementary Fig. 3c), although *SEMA4B* and *SEMA4C* had the highest mRNA levels, but the levels were variable among different GSC lines, which may underlie cell line-specific differences of Plexin-B2 function. The Sema4 genes are also expressed in various combinations by many cell types in mouse brain^32,33^, thus providing additional ligand sources for Plexin-B2. Of note, a recent study demonstrated that Plexin-D1 can function as a mechanosensor in a semaphorin-independent manner^34^, thus, not all functions of Plexins may require activation by a Semaphorin ligand.

### Plexin-B2 KO in GSCs by CRISPR/Cas9

To study the role of Plexin-B2 in GBM invasion, we generated CRISPR/Cas9-mediated Plexin-B2 knockout (KO) in GSCs by targeting the second coding exon of *PLXNB2*. We utilized both plasmid- and lentiviral-based delivery methods to establish clonal and population KO lines, respectively (Supplementary Fig. S3d, e), taking advantage of their complementary features: clonal lines have the benefit of single-cell origin with defined mutations, while population lines maintain more faithfully intratumoral heterogeneity and have stable growth properties, as the cellular stress exerted during clonal selection is avoided. Indeed, we observed high variability of tumor expansion in intracranial transplants of clonal lines derived from the same parental GSCs, we thus conducted the majority of our in vivo assays using population KO lines. We verified the loss of mature Plexin-B2 in all KO lines by WB and IF (Fig. 2b and Supplementary Fig. 3f–h).

We first compared Plexin-B2 KO and control GSCs in standard 2D cell culture conditions but found no apparent differences in cell morphology, proliferation kinetics, and wound closure capacity in the scratch assay (Supplementary Fig. 4a–c). The self-renewal capacity of GSCs in neurosphere formation assays was also not significantly different (Supplementary Fig. 4d).

### Plexin-B2 deletion limits GBM infiltration

To investigate the impact of Plexin-B2 ablation on GBM infiltration in vivo, we engrafted *PLXNB2* KO and control GSCs into the striatum of SCID mice, focusing on the population *PLXNB2* KO lines of SD2 and SD3, which represent mesenchymal and proneural GBM subtypes, respectively (see Supplementary Fig. 1b). As mentioned above, SD2 transplants expanded slower than SD3, but both exhibited wide dissemination; notably, Plexin-B2 KO limited tumor spread in both SD2 and SD3 transplants (Fig. 2c, f). Specifically, whereas in the control SD2 cohort, tumor cells had spread diffusely throughout the striatum and deep into the contralateral hemisphere along the corpus callosum by 147 dpi or 209 dpi, in the Plexin-B2 KO cohort, we observed that tumor cells remained close to the engraftment site, with few cells penetrating into contralateral hemisphere (Fig. 2c, d). Moreover, while invading tumor cells disseminated mainly as individual cells throughout the striatum in the control cohort, they tended to congregate in bundled streams at the tumor edge in Plexin-B2 KO cohort, which was confirmed by quantification showing decreasing GBM cell densities at tumor periphery (Fig. 2c). Hence, Plexin-B2 deletion not only limits GBM spread but also alters invasion patterns. Due to the slow expansion rate of SD2 transplants, no mice died in either control or KO cohort for up to 209 dpi, the endpoint of our study (Fig. 2e).

Similar tumor invasion phenotypes were observed for SD3 transplants: by 29 dpi, tumor cells in the control cohort had penetrated diffusely in the striatum and deep into the contralateral hemisphere along the corpus callosum; but in the KO cohort, the tumor spread was more limited (Fig. 2f, g). Consistent with the reduced spreading, Plexin-B2 deletion in SD3 led to significantly longer survival, with the median survival increased from 50 days in control mice to 74 days in KO mice (Fig. 2h).

We next tested tumor invasion of transplanted GSCs with forced overexpression (OE) of Plexin-B2, and WB indicated that Plexin-B2 levels reached ~1.7- to 2.4-fold in OE cells relative to that in control (see Supplementary Fig. S8a, b)). Interestingly, Plexin-B2 OE also reduced GBM spread, with tumor cells remained congregated at the engraftment site (Supplementary Fig. 5a), suggesting that a fine-tuned and balanced Plexin-B2 activity is required for successful GBM invasion.

### Plexin-B2 deletion shifts the migratory path of invading GBM cells

**Fig. 3:**
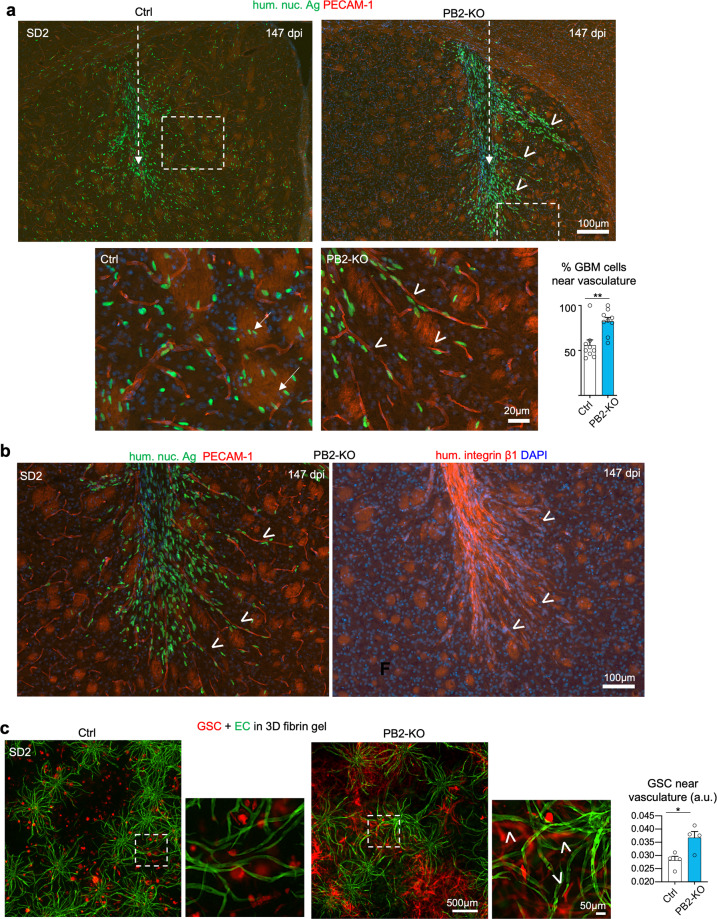
Plexin-B2 deletion shifts preferred migratory path from axon tracts to the microvasculature. **a** Representative IF images of coronal sections of brains with SD2 transplants at 147 dpi (dashed arrows indicate injection tract). While tumor cells (hum. nuc. Ag^+^) in the control cohort had invaded diffusely in the striatum as individual cells, they were largely confined to the transplant site in the PB2-KO cohort, revealing increased aggregation and collective invasion into neighboring brain tissue in bundles (arrowheads). Bottom, enlarged images of boxed areas show that invading GBM cells in control transplant displayed a predilection for striatal fiber tracts (arrows), while PB2-KO cells shifted their preference to perivascular invasion (arrowheads). Note that the nuclear orientation of the migrating PB2-KO cells is closely aligned with vascular axes (PECAM-1^+^). The bar graph shows quantification of the invading tumor cells along vasculature, defined as cells that are one or less than one cell diameter apart from the vessel. *n* = 9 areas from three independent transplants; unpaired *t* test; **P < 0.01. **b** IF images of adjacent brain sections near striatum from mice transplanted with PB2-KO SD3 GSCs at 147 dpi. Left image: at the invasion front, mutant GBM cells showed a clear preference for perivascular invasion (PECAM-1) with nuclear orientation aligned with the vessel axis (arrowheads). Right image: IF for human-specific integrin β1 shows collective chain migration of PB2-KO cells in bundles (arrowheads) that clearly avoided striatal fiber tracts, visible by background fluorescence signals. Note that migrating GBM cells closely adhere to blood vessels at the invasion front (compare images in the left and right panels). **c** Images from 3D vascular network cultures (containing human brain microvascular endothelial cells (GFP^+^), pericytes, and astrocytes) that were seeded with control or PB2-KO SD2 GSCs expressing mCherry. Photos were taken 14 days after cell seeding. PB2-KO cells displayed a bias toward vascular adherence (arrowheads). Quantification on the right indicates the relative abundance of tumor cells that were in close proximity to the vasculature (0–20 µm distance). *n* = 4 independent experiments per group. unpaired *t* test; *P < 0.05.

The altered infiltrative pattern of GBM cells in Plexin-B2 KO transplants suggested a possible change of migratory paths. We, therefore, inspected the nuclear orientation and cell morphology of the invading GBM cells in relation to axon tracts and vascular axes. While in the control transplants, invading tumor cells displayed a preference for axon fiber tracts, they displayed an increased preference for migration along microvasculature in Plexin-B2 KO cohort, with nuclear orientation in alignment with blood vessel axes (Fig. 3a, b and Supplementary Fig. 5b, c). Quantification indicated that up to twice as many tumor cells were found adherent to the microvasculature in KO transplants as compared to controls (82.2% vs. 55.8% for SD2, and 64.0% vs. 33.5% for SD3 transplants, respectively) (Fig. 3a and Supplementary Fig. 5c). Consistently, IF for human integrin β1 outlined the orientation of the migrating GBM cells at the tumor border in close alignment with blood vessel axes in the Plexin-B2 KO transplants, in contrast to the alignment with striatal axon bundles in controls (Fig. 3b and Supplementary Fig. 5b). Furthermore, it also highlighted migratory chains of invading tumor cells along vasculature, reminiscent of migration phenotype observed in Plexin-B2 KO mice, where aberrant chains of migrating neuroprogenitors are found in the olfactory bulb^35^.

To further investigate the preference for the perivascular invasion of Plexin-B2-deficient GSCs, we constructed a 3D culture model of the vascular network in a fibrin matrix, containing human brain microvascular endothelial cells (hBMVECs) labeled with GFP, together with human primary pericytes and astrocytes, which supported lumen formation and vascular stability. We embedded control and Plexin-B2 KO GSCs into the 3D vascular model, and indeed found a significantly higher proportion of KO cells displaying perivascular adherence than control cells, and they also assumed an elongated morphology in alignment with blood vessel axes, recapitulating the in vivo phenotypes (Fig. 3c).

### Plexin-B2 modulates cell–cell adhesion of GSCs

**Fig. 4:**
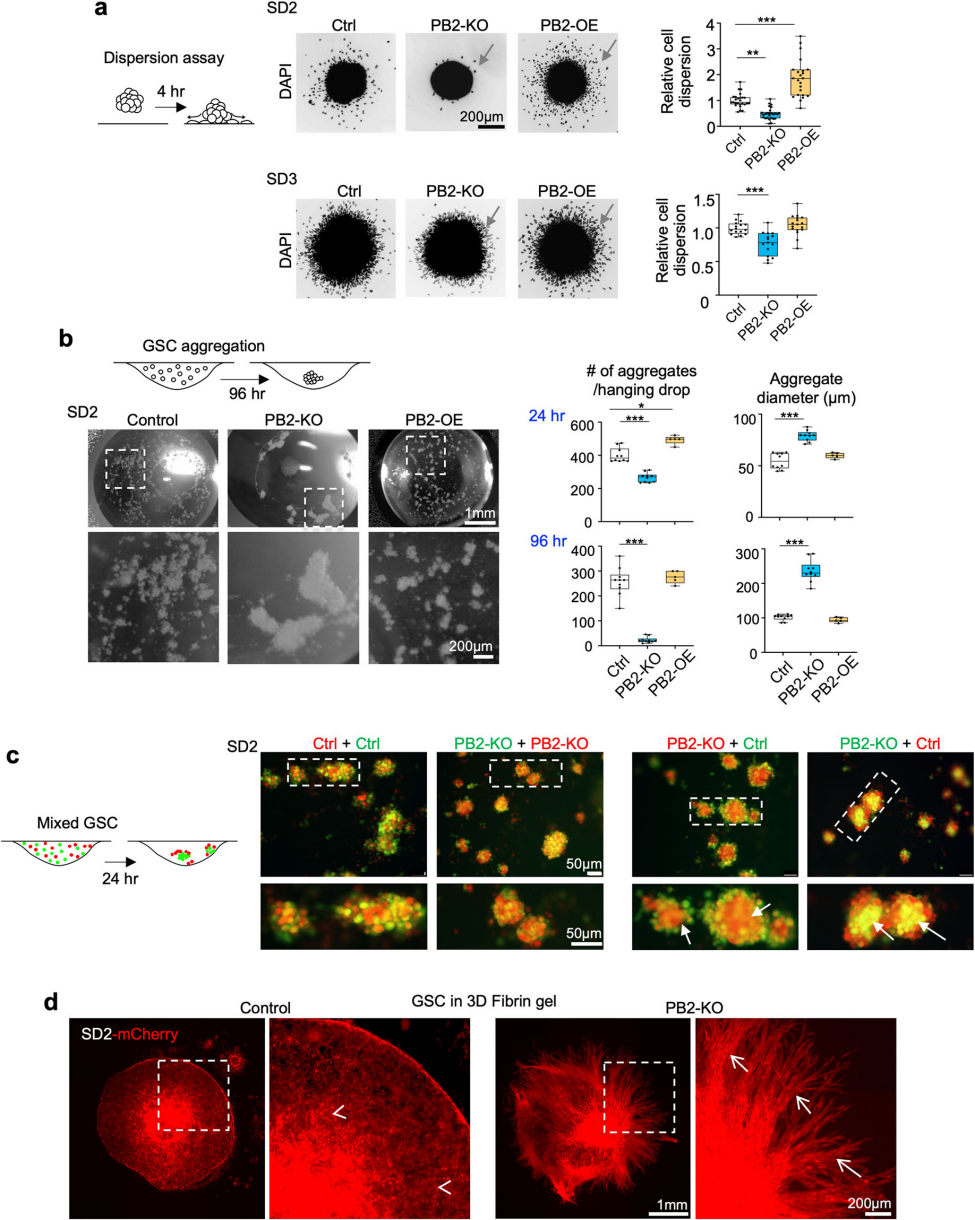
Plexin-B2 lowers cell adhesiveness in GSCs. **a** Left, diagram of cell dispersion assay with 3D aggregates plated on the laminin-coated surface and analyzed after 4 h for dispersion of cell from aggregates. Center, micrographs of DAPI stained aggregates and surrounding dispersed cells (arrows). Right, quantifications of cell dispersion, normalized to mean of the control condition. Box plots and whiskers indicate quartiles, center lines indicate median. *n* = 18–34 spheres per group; one-way ANOVA with Dunnett’s multiple comparison test of each group against control; *P < 0.05, **P < 0.01, ***P < 0.001. **b** Diagram illustrates the hanging drop cell aggregation assay. Photos of drops show aggregates formed by SD2 GSCs with the indicated Plexin-B2 manipulation, 96 h after seeding. Right, quantifications of aggregate numbers and sizes at 24 and 96 h. *n* = 5–11 hanging drops per group; one-way ANOVA with Dunnett’s multiple comparison test of each group against control; *P < 0.05, ***P < 0.001. **c** Left, diagram of differential hanging drop cell aggregation assay with GSCs of different genotypes labeled with green or red CellTracker dyes, and mixed 1:1 before seeding. Right, fluorescence images of SD2 aggregates after 24 h, revealing that PB2-KO cells congregated in the center (arrows), while control cells were mainly at the periphery, illustrating stronger adhesiveness between PB2-KO cells. The mixture of GSCs with identical genotypes (Ctrl + Ctrl, or KO + KO) leads to evenly distributed aggregates. **d** Fluorescence images of SD2 GSCs expressing mCherry that had been embedded as aggregates in 3D fibrin gel matrix. Note the striking differences in the growth/invasion patterns after 27 days: control cells invaded diffusively as individual cells (arrowheads), whereas PB2-KO GSCs invaded the surrounding matrix collectively as fasciculated migration streams (arrows).

We next investigated cellular mechanisms of Plexin-B2 function in GSCs that could affect their invasiveness. As standard 2D cultures or scratch wound assays did not reveal significant changes in Plexin-B2 KO GSCs, we thus turned to assays that incorporate cell–cell/cell–matrix interactions in 3D conditions. The in vivo Plexin-B2 KO phenotypes of tumor cell migration streams suggested increased intercellular adhesiveness. We investigated this possibility by designing a cell dispersion assay to gauge the ability of cells to break away from 3D cell aggregates, which requires lowering intercellular adhesion while gaining traction on 2D laminin-coated surfaces. To this end, GSCs were first allowed to form 3D aggregates, which were then plated onto laminin-coated dishes, and the number of cells dispersing from the aggregates over the course of 2–4 h were analyzed. Indeed, for both SD2 and SD3 GSCs, the dispersion rates were significantly lower for Plexin-B2 KO cells, but higher for Plexin-B2 OE cells when compared to controls (Fig. 4a and Supplementary Fig. 6a). This supports the model that Plexin-B2 enhances invasiveness by promoting dissociation of GSCs from neighboring cells.

We next used a 3D cell aggregation assay to directly assess cell adhesiveness^36^. Dissociated GSCs were seeded in hanging drops at a high density, and the number and size of cell aggregates formed over 24–96 h were quantified. Strikingly, Plexin-B2 KO resulted in fewer but larger cell aggregates for both SD2 and SD3 GSCs; conversely, Plexin-B2 OE led to an increased number of aggregates with comparable sizes at 24 h for both SD2 and SD3, while at 48 h, only SD3 showed an increased number and smaller cell aggregates relative to control (Fig. 4b and Supplementary Fig. 6b). To further examine intercellular adhesiveness, we utilized a differential cell aggregation assay^36^ with GSCs labeled with different dyes. We observed that SD2 GSCs of the same genotype mixed evenly, but when control and PB2-KO SD2 GSCs were mixed together, the PB2-KO cells congregated in the center, and control cells at the periphery, signifying higher cell adhesiveness between Plexin-B2 KO cells (Fig. 4c). Of note, this differential cell adhesion assay was not suited for SD3 GSCs due to their faster aggregation rate, making it difficult to discern fluorescence patterns in larger aggregates (Supplementary Fig. 6c).

Consistent with its role in controlling cell adhesiveness in 3D environment, Plexin-B2 also dictated the mode of cell infiltration from GSC aggregates embedded in 3D fibrin gels. After 27 days of culture, control SD2 GSCs spread diffusely as individual cells toward the periphery, but Plexin-B2 KO cells penetrated the surrounding matrix collectively in streams (Fig. 4d), mirroring the in vivo invasion phenotypes. Taken together, the series of assays lend strong support to the model that Plexin-B2 regulates cell adhesion, which may account for the reduced dispersion rate and increased chain migration pattern for Plexin-B2 KO GBM cells in vitro and in vivo.

### Plexin-B2 influences cell–matrix biomechanical inter**a**ctions and durotaxis behavior

**Fig. 5:**
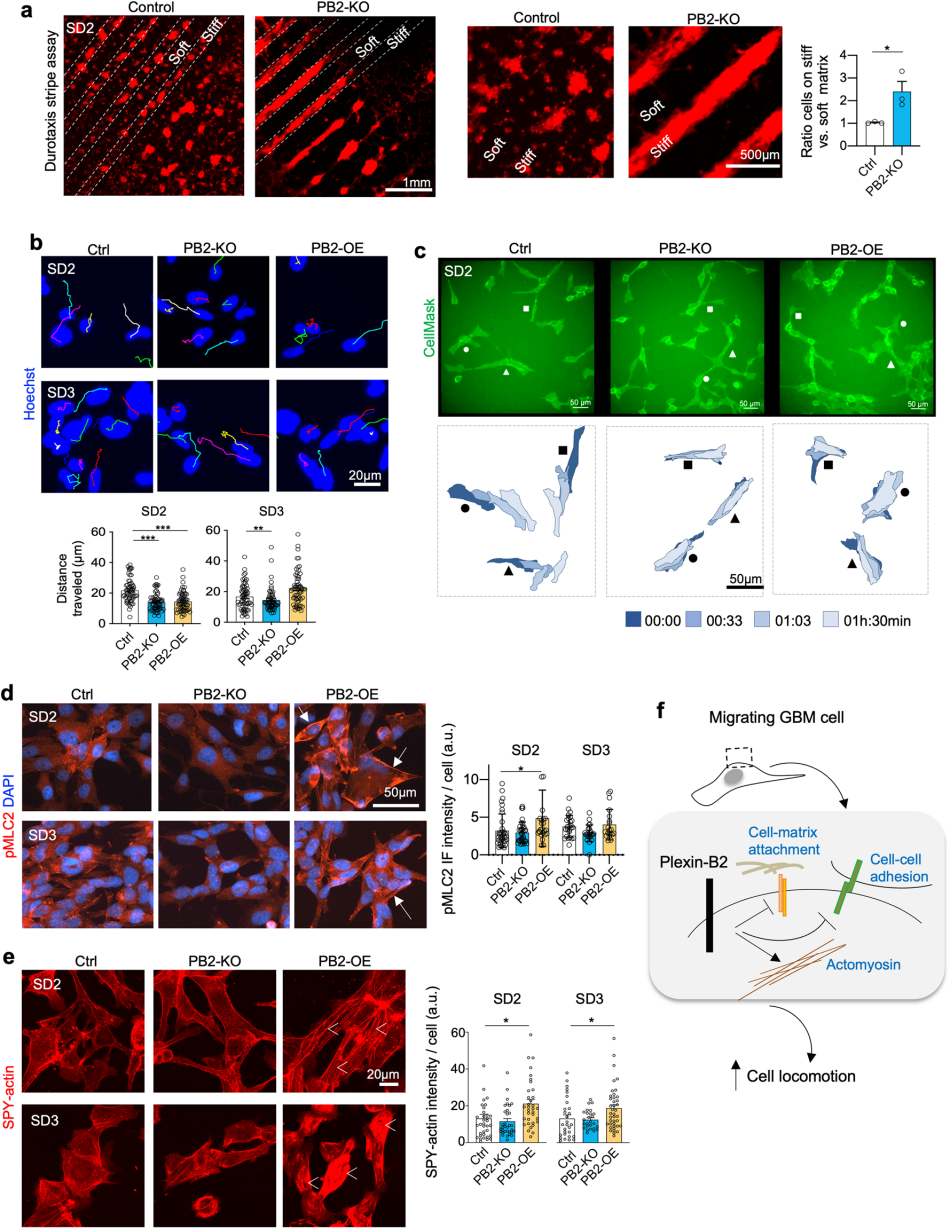
Plexin-B2 counters durotaxis and enhances cell locomotion and actomyosin network. **a** Durotaxis stripe assay. SD2 GSCs expressing mCherry were cultured for 10 days on alternating stripes of soft (~25 kPa) vs. stiff (~30 kPa) PEG substrates. While control GSCs spread on both soft and stiff stripes, PB2-KO cells aggregated only on stiffer stripes. Enlarged images are shown on the right. Quantification measures mCherry fluorescence signals from soft vs. stiff stripes. *n* = 3 independent experiments per group. *P < 0.05, unpaired *t* test. **b** Still frames captured from time-lapse live imaging of GSCs, tracked with Hoechst nuclear dye (colored lines track individual nuclei). Quantifications reveal reduced migration distance over 90 min for PB2-KO GSCs as compared to controls (see Supplementary Videos). SD2 PB2-OE GSCs also had reduced speed of locomotion, but no significant change for SD3 was detected. *n* = 90 tracked nuclei per condition; one-way ANOVA with Dunnett’s multi-comparison test; *P < 0.05; **P < 0.01. **c** Top, still frames from time-lapse live imaging of the indicated SD2 GSCs labeled with CellMask membrane dye. Bottom, three selected cells in each group shown in overlapping contour plots at 30 min-intervals over 90 min. Note the dynamic movement of control cells as compared to both PB2-KO and -OE conditions (see supplementary videos). **d** IF images show increased levels of phospho-myosin light chain 2 (pMLC2, arrows) in PB2-OE SD2 GSCs as compared to control cells. Right, quantifications show increased levels of IF intensity for pMLC2 per cell (corrected total fluorescence quantification; a.u., arbitrary unit). *n* > 30 cells for each condition. One-way ANOVA with Dunnett’s multi-comparison test; *P < 0.05. Differences for SD3 GSC did not reach statistical significance. **e** Live-cell imaging of GSCs stained with F-actin dye SPY-actin. Overexpression of PB2 in SD2 and SD3 GSCs leads to increased actin filament formation (arrowheads). Quantification indicates corrected total cell fluorescence; a.u., arbitrary unit. *n* > 30 cells for each condition. One-way ANOVA with Dunnett’s multi-comparison test; *P < 0.05. **f** Working model. Plexin-B2 controls actomyosin dynamics and interactions of invading GBM cells with neighboring cells and matrix substrates along migratory paths.

Invading tumor cells are exposed to a changing mechanical environment, hence migrating GBM cells need to adjust not only adhesiveness to neighboring cells but also their mechanical interaction with matrix substrates along the migratory paths that may have distinct physical properties. We, therefore, studied the impact of Plexin-B2 on the biomechanical interaction of migrating GSCs with substrates of different stiffness. Most adherent cells migrate towards higher substrate stiffness, a behavior known as durotaxis^5^. This creates a mechanical challenge for GBM cells that attempt to dissociate from the stiff tumor bulk and invade softer brain parenchyma^37^. To test if Plexin-B2 would influence durotactic behavior, we seeded dissociated GSCs on alternating stripes of the soft and stiff substrate and examined their spread. Strikingly, after 10 days of culture, whereas control SD2 GSCs were found to spread on both soft and stiff stripes, Plexin-B2 KO cells congregated only on stiffer stripes and did not spread significantly on the softer stripes (Fig. 5a). A similar, but the weaker effect was also observed for SD3 GSCs (Supplementary Fig. 6d). As Plexin-B2 is highly expressed in GSCs, our data suggest that Plexin-B2 counters durotactic tendency and bestows the capability to migrate regardless of substrate stiffness. This capability may be critical for GBM cells to break away from stiff tumor bulk and initiate invasion into softer brain tissues.

### Plexin-B2 influences cell locomotion and actomyosin network distribution

Invading GBM cells can successfully negotiate tight interstitial spaces by assuming a fusiform morphology and adhering closely to migratory surfaces. To examine cell locomotion in dependence of Plexin-B2, we performed time-lapse live-cell imaging of GSCs. We assayed cell movement by tracking either the nuclei or the cell contours using the CellMask membrane dye, which showed that SD2 and SD3 PB2-KO GSCs displayed reduced cell locomotion compared to control cells (Fig. 5b, c and Supplementary Videos 1–4). Notably, SD2 Plexin-B2 OE GSCs also moved slower than control GSCs, indicating that forced overexpression of Plexin-B2 also negatively impacted their locomotor behavior.

To examine whether Plexin-B2 affects actomyosin contractility, we examined the expression of phospho-myosin light chain II (pMLC2), an initiator of actomyosin contraction^38^. Indeed, IF showed that Plexin-B2 OE GSCs exhibited an increased cortical network of pMLC2, whereas Plexin-B2 KO cells showed a trend of reduction relative to control (Fig. 5d). We further performed live-cell imaging of GSCs stained for actin filament, which confirmed increased F-actin formation in PB2-OE GSCs (Fig. 5e). Altogether, Plexin-B2 enhances GBM invasiveness in the complex mechanical environment by lowering cell adhesiveness, bestowing the capability to spread on soft substrates (i.e., countering durotaxis), and adjusting actomyosin network distribution and cell locomotion (Fig. 5f).

### Plexin-B2-associated transcripti**o**nal signatures point to biomechanical functions

**Fig. 6:**
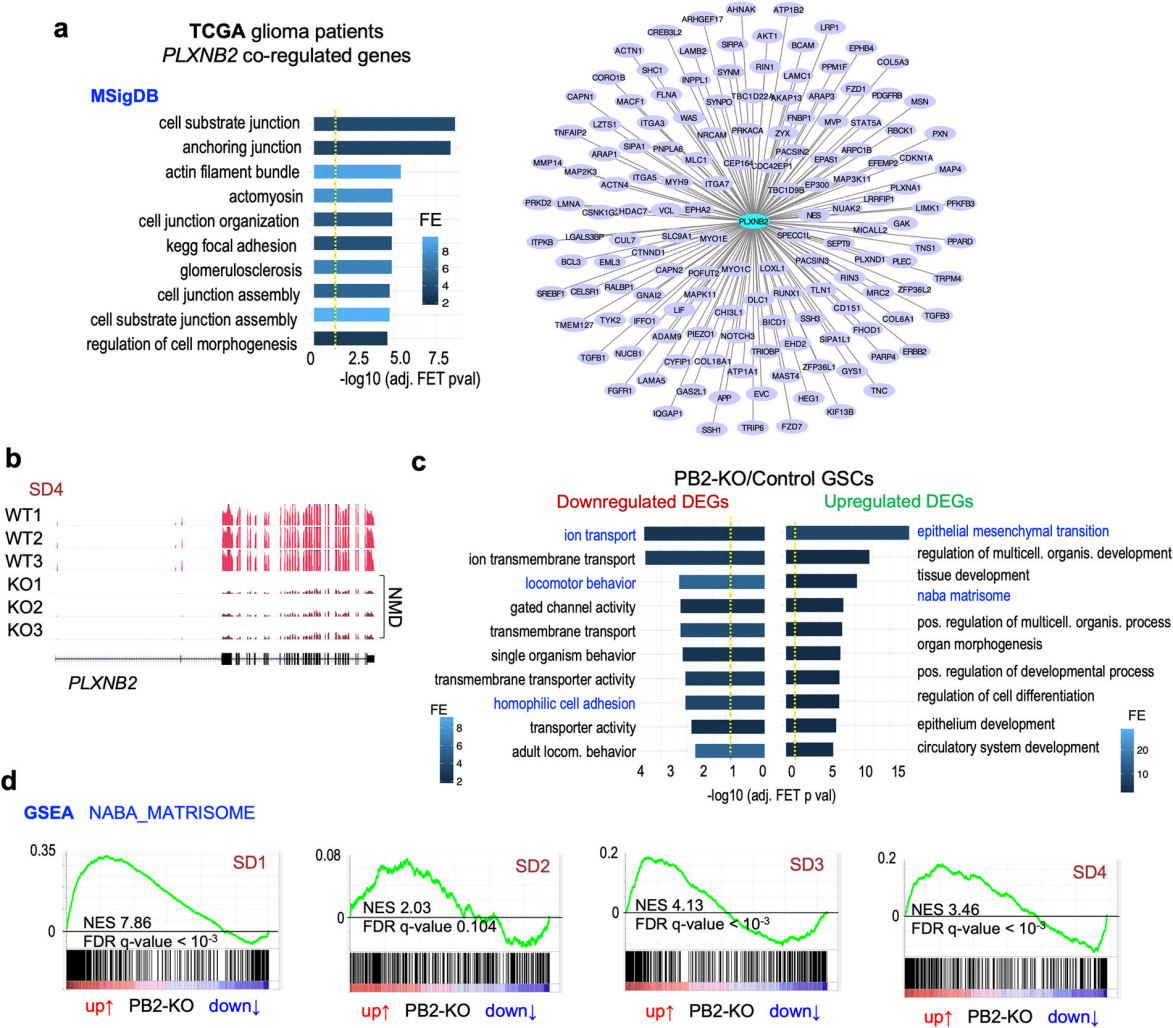
Gene expression analyses link Plexin-B2 to biomechanical gene signatures in GBM. **a** Genes that are correlated with *PLXNB2* expression levels in GBM patients (Spearman correlation FDR < 10%, TCGA database) are enriched for pathways concerning cell adhesion, cell-substrate junctions, and actomyosin. Left, top 10 significant functional terms (MSigDB) are shown. Right, diagram of connectivity of coregulated genes with *PLXNB2* in human GBMs, filtered for genes involved in cell adhesion, motility/invasion, and EMT. **b** Examples of RNA-seq read tracks for *PLXNB2* mRNA in three GSC clonal lines of wild-type and PB2-KO genotypes from SD4 GSCs. Frameshift mutations in the PB2-KO lines lead to the reduction of *PLXNB2* mRNA levels by nonsense-mediated decay (NMD). **c** Pathway enrichment analyses of up- and downregulated DEGs in response to Plexin-B2 KO (union of all DEGs from four GSC lines; FDR < 20%). Top ten significantly enriched terms (MSigDB) are shown. Dashed yellow lines denote −log10 (adjusted *P* value) = 0.05. **d** GSEA of expression changes in PB2-KO vs. control GSCs shows enrichment of Matrisome gene set (Naba et al.^39^) for all four GSC lines. The sinusoidal shapes of enrichment scores (green line) indicate enrichment of both up- and downregulated genes.

To examine Plexin-B2-associated molecular signatures in GBM, we first turned to the TCGA (The Cancer Genome Atlas) GBM patient database and extracted genes with expression levels correlated with *PLXNB2* levels in GBM (Spearman’s rho > 0, FDR < 10%). Systems biology analysis showed that *PLXNB2*-correlated genes were significantly enriched for cell adhesion, cell-substrate junction, and actomyosin pathways (Fig. 6a), thus providing molecular support for the link of Plexin-B2 with cell biomechanics.

We next examined gene expression changes in GSCs in response to Plexin-B2 deletion by performing RNA-seq analysis. The quality of the RNA-seq data was first confirmed by the reduced levels of mutant *PLXNB2* transcripts in PB2-KO GSCs with frameshift mutations due to nonsense-mediated decay (Fig. 6b). We identified differentially expressed genes (DEGs, adj. *P* < 0.01) in Plexin-B2 KO GSCs as compared to control counterparts, which ranged from 7 to 950 genes for lines SD1–SD4 (Supplementary Fig. 7a). The overlap of different sets of DEGs was small, reflecting cell line-specific transcriptional responses to the biomechanical changes upon Plexin-B2 deletion. Pathway enrichment analyses of up- and downregulated DEGs revealed their involvement in locomotor behavior, epithelial-mesenchymal transition (EMT), and the Matrisome, which is comprised of a collection of extracellular matrix (ECM) and cell adhesion proteins^39^ (Fig. 6c). Ion transport genes were also enriched among DEGs, which can support cell locomotion by regulating hydrodynamic pressure inside cellular compartments^3^. Concordantly, gene set enrichment analysis (GSEA), which matches ranked gene lists to functional gene sets, revealed enrichment of Matrisome gene set (in both up- and downregulated spectrum in response to Plexin-B2 KO) in all four GSCs, including all Matrisome subsets (Fig. 6d and Supplementary Fig. 6b). Taken together, transcriptional profiling of Plexin-B2 KO vs. control GSCs revealed similar findings as the analyses of *PLXNB2*-coregulated genes in TCGA patient dataset, both pointing towards a strong association of Plexin-B2 with gene pathways that concern cell locomotion and biomechanical properties.

### Plexin-B2 engages the intracellular GAP domain to regulate cell biomechanical properties

Plexins can signal through Ras and/or Rho small G proteins to regulate cytoskeletal dynamics^12,40^. The highly conserved intracellular Ras-GAP domain of Plexins has been reported to inactivate small G proteins such as R-Ras, M-Ras, and Rap1/2^41–43^, whereas the C-terminus of Plexin-Bs contains a PDZ-binding motif for docking of PDZ-Rho-GEF and LARG, two guanine nucleotide exchange factors (GEFs) that can activate RhoA^44^. In addition, Plexins also contain a Rho-binding domain (RBD) where Rac1 or Rnd1/2/3 can bind, presumably to modulate Plexin activity^41,45,46^.

**Fig. 7:**
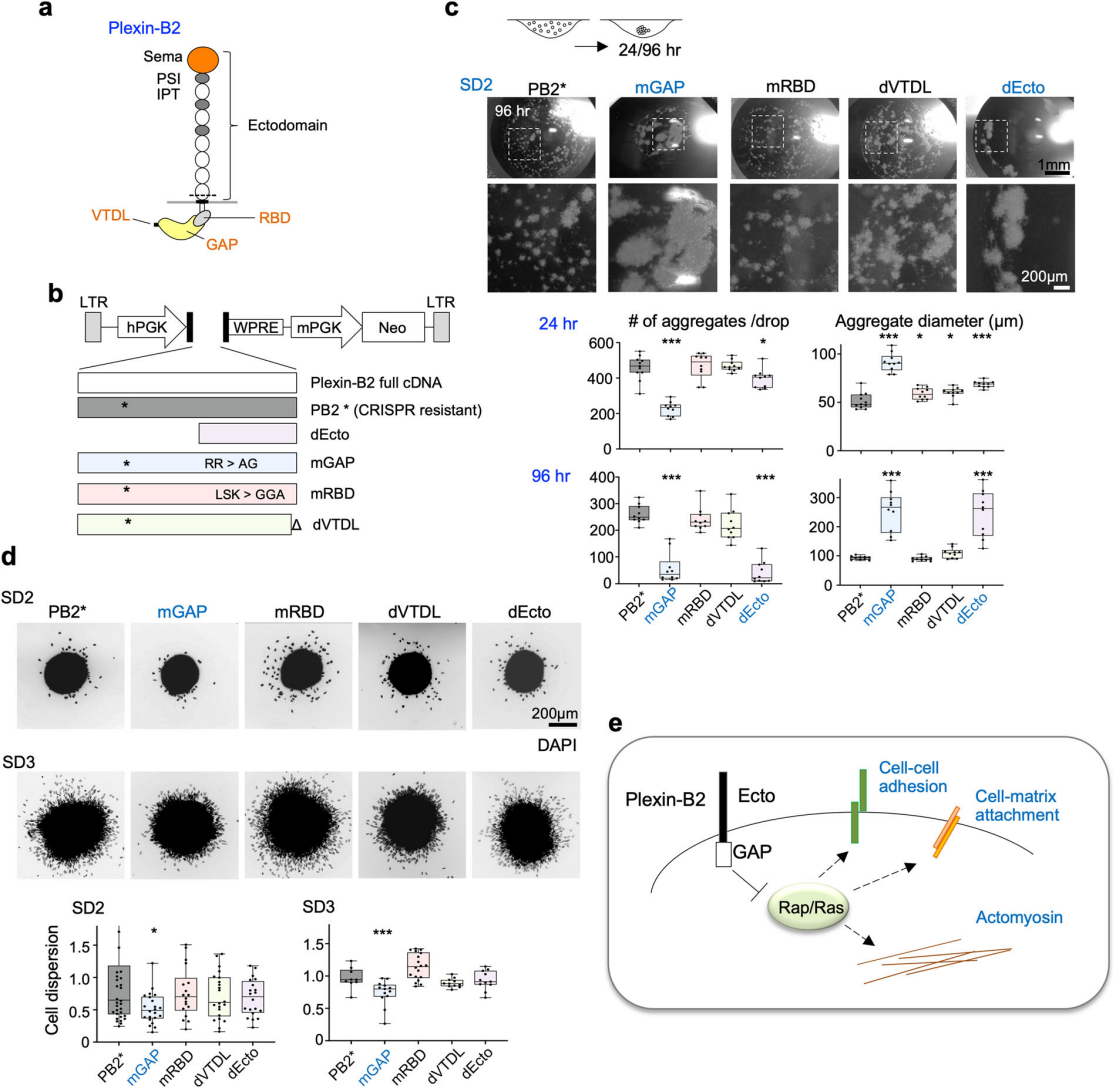
Plexin-B2 signaling engages the Ras-GAP domain. **a** Cartoon of Plexin-B2 domain structure. Cell membrane shown as a gray bar; dashed line indicates proteolytic cleavage into α and β chains. Sema, Sema domain; PSI, plexin-semaphorin-integrin domain; IPT, Ig-like fold shared by plexins and transcription factors; RBD, Rho-binding domain; GAP, GTPase activating protein; VTDL, PDZ-domain binding site formed by four C-terminal amino acids for docking of PDZ-Rho-GEF or LARG proteins. **b** Diagrams of lentiviral vectors encoding cDNA of wild-type or signaling mutants of Plexin-B2. PB2* is CRISPR-resistant cDNA, due to synonymous mutations (X) at sgRNA target site. dEcto lacks extracellular domain, mGAP has mutated GTPase activation domain, mRBD has mutated Rho-binding domain, and dVTDL has deleted PDZ-binding motif. **c** Cell aggregation assay in hanging drops used for Plexin-B2 (PB2) rescue experiments. Photos and enlargements show aggregates formed from GSCs with the indicated Plexin-B2 mutations in hanging drops after 96 h. Quantifications of aggregate numbers and sizes in hanging drops at 24 or 96 h are shown below. The mGAP and dEcto Plexin-B2 mutants failed to rescue the KO phenotype (marked as blue). *n* = 5–11 drops per group; one-way ANOVA with Dunnett’s multiple comparison test of each group against SD2-Ctrl; *P < 0.05, ***0.001. **d** Left, representative micrographs of cell dispersion assay with GSCs expressing mutant Plexin-B2 rescue constructs. Dispersed cells from aggregates were visualized at 4 h with DAPI nuclear stain. Bottom, quantifications show the number of nuclei detached from spheres normalized to mean in the control condition. The PB2 mGAP mutant failed to rescue the KO phenotype (marked as blue). Box plots and whiskers indicate quartiles, center lines indicate median. *n* = 18–34 spheres per group; one-way ANOVA with Dunnett’s multiple comparison test of each group against control; *P < 0.05, ***P < 0.001. **e** Model depicting Plexin-B2 signaling through Ras-GAP domain to affect cell biomechanics during GBM invasion.

To pinpoint the Plexin-B2 domains that are involved in controlling cell biomechanics of GSCs, we conducted structure-function analyses by introducing into Plexin-B2 KO GSCs a series of Plexin-B2 signaling mutants (with additional mutations at sgRNA target site, rendering them resistant to CRISPR-mediated KO) for rescue experiments (Fig. 7a, b and Supplementary Fig. 8a).

Using the hanging drop cell aggregation assay, we found that Plexin-B2 with mutations in the Ras-GAP domain (mGAP) or deletion of the extracellular domain (dEcto) generally failed to rescue the KO phenotype, evidenced by the larger cell aggregates of these mutant lines (Fig. 7c and Supplementary Fig. 8c). This indicates an essential role of these two domains in lowering cellular adhesion. Of note, we observed a certain degree of variability, particularly at 96 h, and it remains possible that the GAP dead Plexin-B2 might still partially substitute for Plexin-B2 function in some cases, perhaps by engaging other parts of the Plexin-B2 intracellular domain, such as the VTDL PDZ-binding motif.

In the cell aggregate dispersion assay, only the Ras-GAP, but not the ectodomain appeared to be essential, as fewer cells dispersed from 3D cell aggregates with mGAP (Fig. 7d). The rescue by the dEcto mutant indicated a possible semaphorin-independent function of Plexin-B2 for mediating cell dispersion, for which the intracellular domain might be sufficient. In this context it interesting to note that dEcto mutants of Plexin-As can activate cell collapse^47,48^ In sum, our structure-function analysis points towards a central role of the Ras-GAP activity of Plexin-B2 in controlling cell biomechanical properties (Fig. 7e).

### Plexin-B2 influences Rap1/2 and YAP a**c**tivity in GSCs

Given the critical role of the Ras-GAP domain of Plexin-B2 in mediating adhesive properties and cell dispersion, we further examined the effects of Plexin-B2 on Ras activation, focusing on the putative targets Rap1 and Rap2^43^. We confirmed by WB that Rap1 and Rap2 were expressed in GSCs, and Plexin-B2 KO or OE did not appear to change total protein levels of Rap1/2 (Supplementary Fig. 9a; “input”). We then measured levels of active Rap1 and Rap2 (i.e., GTP-bound) in 3D GSC aggregates that were briefly stimulated by forskolin to enhance baseline Rap1/2 activation^49^. Plexin-B2 KO resulted in increased levels of active Rap1 and Rap2 in both SD2 and SD3, albeit more consistently in SD2. Unexpectedly, Plexin-B2 OE did not decrease levels of active Rap1/2 in SD2, and even caused higher levels in SD3 GSCs (Supplementary Fig. 9a), suggesting that Plexin-B2 may not be sufficient to decrease Rap1/2 activity and a not yet understood complexity of engagement of Rap1/2 and perhaps other Ras GTPases by Plexin-B2 in a cell line-specific manner. It should be noted that it remains possible that changes of Rap1/2 activation may be independent of Plexin-B2 GAP activity, but due to alterations in other components in response to Plexin-B2 manipulations. Future assays with improved sensitivity for Rap activity levels including signaling mutants will be needed to resolve these questions.

As a recent study had shown that Rap2 can relay information of substrate rigidity to mechanosensitive cellular activity through the transcription factors YAP/TAZ^50^, we tested whether Plexin-B2 activity would affect nuclear localization of YAP/TAZ. We observed a trend of increase in the nuclear localization of YAP/TAZ in Plexin-B2 OE GSCs, however, results in PB2-KO cells were variable, with SD3 Plexin-B2 KO showing a decrease, but SD2 Plexin-B2 KO cells a slight increase in the nuclear localization of YAP/TAZ (Supplementary Fig. 9b). Thus, while this is interesting, given the variability in different lines, the development of additional assays and approaches is needed to examine the link between Plexin-B2 and YAP/TAZ activity.

## Discussion

Diffuse infiltration is a pathological hallmark of GBM^51^, thus identifying the underlying cellular and molecular mechanisms is of paramount importance. Here, we highlighted complex physical interactions of invading tumor cells with their environment, including neighboring cells as well as the matrix substrate. Through a series of in vitro assays designed to understand how GBM cells operate to gain invasiveness, coupled with in vivo orthotopic transplant studies, we unraveled three underlying biomechanical mechanisms by which the guidance receptor Plexin-B2 exerts its function to promote GBM invasion.

First, we demonstrated that Plexin-B2 lowers cellular adhesion, a critical step for GBM cells to detach from tumor bulk and initiate invasion. The enhanced cellular aggregation of Plexin-B2-deficient GSCs echoes the ectopic aggregation of cerebellar granule cells in the developing cerebellum in Plexin-B2 KO mice^20^. It is intriguing that Plexin-B2 functions to reduce cellular adhesion in the context of GBM invasion, as Plexins were originally described as homophilic adhesion molecules^52^, and in a neuroblastoma model, Plexin-As have been shown to act as pro-adhesion molecules^53^. Future analyses are needed to resolve these divergent effects, which may be related to different cancer models or differences between Plexin subclasses.

Second, Plexin-B2 dictates how GSCs respond to different substrate stiffness. In a stripe assay, we showed that Plexin-B2 KO GSCs congregated mainly on stiff substrate, while control GSCs were able to spread on both soft and stiff substrates. This is an important observation with clinical implications: as GBM progresses, tumor mass gradually stiffens^7^; yet most normal cell types display durotactic behavior. Hence, GBM cells must find a way to counter durotactic tendency, which may be bestowed by Plexin-B2 upregulation. However, it remains to be determined how Plexin-B2 enables GBM cells to invade softer brain parenchyma, which may be linked to enhanced cytoskeletal contractility or strengthened substrate anchorage to gain sufficient traction forces.

Third, Plexin-B2 facilitates cytoskeletal dynamics, demonstrated by time-lapse videography of cell locomotion, cortical actomyosin network, and molecular signatures. Our finding also agrees with an earlier report on actomyosin contraction as a mediator of plexin function in migrating dendritic cells^54^. Interestingly, Plexins are known for growth cone collapse upon exposure to semaphorins, which is attributed to F-actin depolymerization. It is possible that the acute collapse response might be different from the effect of sustained Plexin-B2 activation from forced overexpression on actomyosin network redistribution, which may occur via a semaphorin-independent mechanism, fitting with our observation that dEcto Plexin-B2 can rescue cell dispersion (see Fig. 7d). Cell intrinsic biomechanical properties are essential for migrating cells to negotiate tight interstitial spaces while maintaining traction on substrate surfaces; in this context, balanced Plexin-B2 activity appeared to be crucial, as both Plexin-B2 deficiency or forced overexpression impeded GBM spread in intracranial transplants. This echoes the requirement for balanced levels of semaphorin/plexin activity during neurodevelopment, where both loss- and gain-of-function conditions can result in similar axon guidance defects^55^.

Our previous analysis of GBM gene expression databases showed that high Plexin-B2 transcription in GBM is associated with poor patient survival^22^; in this study, we observed that forced overexpression of Plexin-B2 impeded GBM infiltration. These data may not be in conflict with one another, as GBM cells may be able to achieve optimal expression levels of Plexin-B2 protein required for desired biomechanical plasticity through feedback regulatory mechanisms onto the endogenous *PLXNB2* promoter or by regulation of mRNA translation, which contrasts with our current experimental paradigm of forced lentiviral overexpression of Plexin-B2 cDNA under a constitute promoter without regulatory sequences in 5′ and 3′UTRs.

To reveal the biomechanical functions of Plexin-B2, our studies underscored the importance of employing assays designed to reflect biomechanical challenges in 3D conditions, as conventional 2D culture assays did not reveal phenotypic changes. Hence, a fundamental function of Plexins lies in the regulation of cellular contacts, likely involving paracrine activation through semaphorins. Alternative activation of Plexin-B2 may also be possible through a potential semaphorin-independent function as a mechanosensor^34^. It has also to be considered that the role of Plexin-B2 as angiogenin entry receptor may contribute to glioma growth^23^, but our functional data on the importance of the intracellular Ras-GAP domain of Plexin-B2 in mediating biomechanical properties of GSCs makes this less likely.

Plexin-B2 ablation shifted preferred migratory paths of invading GBM cells from axon fiber tracts to perivascular routes. In this context, it is noteworthy that the basal lamina that envelopes brain microvasculature has a relatively high stiffness^56^, thus a bias toward perivascular migration might reflect the durotactic preference of Plexin-B2-deficient GBM cells. Future studies are needed to address additional chemical and physical attributes of migration surfaces that dictate the choice of preferred migratory paths. It is also important to be mindful of potential stage-dependent invasion behaviors: in our previous study of early tumor spread of SD2 transplants, we found a significant vascular association of tumor cells at 14 dpi, which appeared to be reduced by Plexin-B2 knockdown^22^, while in this study with more advanced stages of GBM spread (i.e., ~150 to 200 dpi), we found that infiltrating GBM cells exhibited a predilection for axon tracts. Future studies will also be needed to explore whether Plexin-B2 manipulation in GBM cells affects tumor vascularization patterns.

The high intertumoral heterogeneity of GBM is reflected in the distinct responses of different patient-derived GSC lines to Plexin-B2 manipulation in regard to phenotypical characteristics, differentially regulated genes, and Rap1/2 activation. Nevertheless, our data also underscored functional convergence of Plexin-B2 signaling on cell locomotion, cell adhesiveness, and matrisome regulation in different GSC lines. From a therapeutic point of view, exciting new strategies to block plexin function, such as cyclic peptides^57^ and function-blocking antibodies against semaphorins^58^ are worthwhile for future exploration.

We identified that engagement of the Ras-GAP domain is critical for Plexin-B2-mediated cellular biomechanics, a finding that aligns with our recent phylogenetic analyses that revealed the Ras-GAP domain as the best-conserved part of Plexins throughout animal evolution^59^. Developing specific small molecule inhibitors against the Ras-GAP domain of Plexin-B2 (not yet available) thus represents another promising direction to inhibit GBM spread. Future studies are also needed to delineate the link of Plexin-B2, Rap or other Ras GTPases, and YAP/TAZ in regulating mechanosensitive cellular activity.

In summary, our studies highlight the complexity of tumor invasion and the importance of physical interactions of invading GBM cells with the environment that are dictated by the biomechanical properties controlled by proper levels of Plexin-B2 activity. Our data support the model that Plexin-B2 is usurped by GBM cells to enhance invasiveness, counter durotaxis, negotiate vascular vs. axonal substrates, and respond to different properties of the substrate they mobilize on. The new understanding of cell biomechanics can open up new avenues to curb GBM invasion.

## Methods

### Mice

Adult male immunocompromised SCID mice (IcrTac:ICR-*Prkdc*^scid^) for intracranial transplantation studies were purchased from Taconic Biosciences. All animal procedures were performed in accordance with protocols approved by the Institutional Animal Care and Use Committee (IACUC) of Icahn School of Medicine at Mount Sinai.

### Human GBM cell lines

De-identified human GBM stem cell (GSC) lines SD1–SD4 had been established from resected tumor tissue of GBM patients at the University of California, San Diego by Dr. Kesari and Dr. Pingle^22^. GSCs were propagated in neural stem cell media (Neurocult NS-A proliferation kit (human), Stemcell Technologies), supplemented with bFGF (10 ng/ml; Peprotech), EGF (20 ng/ml; Peprotech), Heparin (0.0002%; Stemcell Technologies), and penicillin–streptomycin (1:100; Gibco), and maintained as adherent cultures on culture dishes coated with laminin (10 µg/ml in PBS; 1 h at 37 °C; Invitrogen). For passaging, cells were dissociated with Accutase (BD Biosciences). GSC lines were authenticated by WB and RNA sequencing by analysis of the distinctive expression patterns of EGFR, PDGRA, and MET. The cell lines in this study were tested for mycoplasma contamination and viral infections with the IMAPCT I PCR profile assay (IDEXX BioAnalytics) and found negative.

### Intracranial GCS transplants

For intracranial transplantation, 1 × 10^5^ GSC in 2 µl Opti-MEM (Gibco 31985070) were stereotactically injected into the striatum (Paxinos coordinates: +2 mm lateral, −0.5 mm AP, −3 mm vertical) of adult immunocompromised ICR-SCID mice (IcrTac:ICR-*Prkdc*^scid^; Taconic Biosciences). For survival studies, mice were euthanized when neurological symptoms appeared, and Kaplan–Meier survival curves were calculated with GraphPad PRISM software. Histological analysis of intracranial tumors was performed by immunofluorescence.

### Immunofluorescence (IF) of brain sections and of cultured cells

Mice carrying intracranial GSC transplants were intracardially perfused with PBS, followed by 4% paraformaldehyde (PFA) in PBS. Brains were dissected and post-fixed overnight in 4% PFA/PBS at 4 °C, then cryoprotected by successive incubations overnight in 12.5% and 25% sucrose/PBS at 4 °C. Brains were embedded in TissueTek OCT compound (Sakura), and tissue blocks were frozen on dry ice and stored at −80 °C. Cryostat sections were cut at a thickness of 25 µm and stored as floating sections in PBS at 4 °C. For immunofluorescence staining, sections were blocked for 1 h in blocking buffer (PBS with 5% donkey serum and 0.3% Triton X-100), then incubated at 4 °C overnight with primary antibodies in antibody dilution buffer (PBS with 1% BSA and 0.3% Triton X-100), followed by staining with Alexa secondary antibodies (Jackson ImmunoResearch) for 2 hr, and nuclear counterstaining with DAPI (Invitrogen). Sections were washed in PBS and mounted with Fluoromount G (Southern Biotech) on glass slides. Images were captured with a Zeiss AXIO Imager.A2 microscope with AxioCamMRc camera and AxioVision software.

Quantification of GBM cell scattering was performed by drawing on micrographs in ImageJ a contour of the tumor core as the region with the highest continuous cell density. This shape was expanded four times, with each contour being ×1.5 the previous size. Cells were counted within each ring between the contours. Cell density was normalized to the density within the tumor core.

For IF of cultured cells, GSC were seeded in laminin-coated chamber slides (Thermo Fisher) at low density and fixed after appropriate culture period (>24 h) with 3.7% formaldehyde in PBS at room temperature for 10 min. Cells on chamber slides were then blocked for 1 h in blocking buffer (PBS with 5% donkey serum and 0.3% Triton X-100), and incubated overnight at 4 °C with primary antibodies in antibody dilution buffer (PBS with 1% BSA and 0.3% Triton X-100), followed by staining with Alexa-conjugated secondary antibodies (Jackson ImmunoResearch) for 2 h. Isotype control staining was conducted by substituting primary antibodies with purified whole IgG from the same species (Jackson ImmunoResearch) at the corresponding dilution. Staining for F-actin was performed with rhodamine-phalloidin (Invitrogen), and nuclear counterstaining with DAPI (Invitrogen).

### Western blotting

For protein detection by western blotting, lysates of cells were prepared with RIPA buffer (Sigma) containing protease and phosphatase inhibitors. Lysates were resolved by SDS-PAGE on 4–12% NuPAGE Bis-Tris gradient gels (Invitrogen) and transferred onto nitrocellulose membranes with the Novex gel system (Invitrogen). Membranes were incubated at 4 °C overnight with primary antibodies and then for 1 hr with secondary donkey antibodies IRDye 680 and 800 (Li-Cor) for detection of bands with the Odyssey Infrared Imaging System (Li-Cor). Uncropped images of Western blot membrane scans are shown in Supplementary Figs. 10 and 11.

### Primary antibodies

Antibodies used for western blot analysis (WB) or immunofluorescence (IF) were as follows:

anti-β-actin, Sigma A1978 (host species: mouse), dilution 1:10,000 for WB

anti-EGFR, Cell Signaling Technology 4267 (rabbit), 1:1000 for WB

anti-human nuclear antigen (HNA), Millipore MAB1281 (mouse), 1:250 for IF

anti-integrin β1 (human-specific clone TS2/16), Santa Cruz sc-53711 (mouse), 1:300 for IF

anti-laminin, Millipore MAB1905 (rat), 1:200 for IF

anti-MBP, CST 78896 (rabbit), 1:100 for IF

anti-MET, Cell Signaling Technology 8198 (rabbit), 1:1000 for WB

anti-pMLC2 (Ser19), Cell Signaling Technology 3671 (rabbit), 1:100 for IF

anti-PDGFRα, Cell Signaling Technology 3174 (rabbit), 1:1000 for WB

anti-PECAM-1/CD31, BD Biosciences 553370 (rat), 1:300 for IF

anti-Plexin-B1, R&D systems AF3749 (goat), 1:400 for WB

anti-Plexin-B2, extracellular domain, R&D systems AF5329 (sheep), 1:400 for WB, 1:100 for IF

anti-Plexin-B2, intracellular domain, Abcam ab193355 (rabbit), 1:1000 for WB

anti-Plexin-B3, R&D systems AF4958 (sheep), 1:400 for WB

anti-Sema4B, Proteintech 16934-1-AP (rabbit), 1:400 for WB

anti-Sema4C, LSBio C150056 (sheep), 1:400 for WB

anti-YAP/TAZ, Santa Cruz sc101199 (mouse), 1:100 for IF.

For isotype control IF staining, we used serum-purified IgG fractions from the same host species as the primary antibody at similar concentrations (Chromepure IgG, Jackson ImmunoResearch).

### CRISPR/Cas9 p**l**asmid and lentiviral vectors for Plexin-B2 KO

For plasmid-based CRISPR/Cas9 mutation of *PLXNB2*, the plasmid pX459-sgPLXNB2 was generated, which expresses Cas9 and a small guide RNA against the second coding exon of *PLXNB2* (sgPB2, GTTCTCGGCGGCGACCGTCA), using pX459 as backbone^60^.

For lentivirus-based CRISPR/Cas9 mutation, the plasmid pLentiCRISPRv2-sgPLXNB2 expressing sgPB2 was generated, using pLentiCRISPRv2 as backbone^61^. A plasmid pLentiCRISPRv2-sgEGFP expressing sgRNA against an EGFP coding sequence was used to generate control cells (sgEGFP, GGGCGAGGAGCTGTTCACCG). Lentiviral particles were produced by co-transfecting 293T cells with lentivirus plasmid, envelope plasmid pMD2.G, and packaging plasmid psPAX2 (Addgene 12259 and 12260; deposited by Didier Trono, EPFL Lausanne). Cell supernatants were collected 48 h after transfection and viral particles were concentrated by ultracentrifugation before being used for infection of cells overnight.

### Generation of Plexin-B2 KO cells

For plasmid-based CRISPR/Cas9 mutation, a cell suspension of GSC was electroporated with the Neon transfection system (Invitrogen) using the following parameters: 1 × 10^6^ cells/cuvette, 10 µg of plasmid DNA of pX459-sgPLXNB2 (see above), 3 pulses for 10 ms at 1300 V. Cells were seeded after electroporation on laminin-coated six-well plates at a density of 10^6^ cells per well, and the medium was changed after 24 h. Two days later, cells were harvested and seeded at a concentration of 10 cells/ml in ultra-low-attachment 96-well plates (Corning 3474), using 100 µl per well. After 10 days, clonal spheres in wells were visually identified and transferred to laminin-coated 24-well plates, and then expanded and analyzed by sequencing for mutations at the sgPLXNB2 target site and by western blot for Plexin-B2 protein expression. Clonal lines that did not carry mutations of *PLXNB2* were used as matching controls in subsequent experiments.

For lentivirus-based CRISPR/Cas9 mutation, adherent cultures of GSC were infected overnight with LentiCRISPRv2-sgPLXNB2 and polybrene (8 µg/ml; Sigma) in six-well dishes (2 × 10^5^ cells per well). Starting two days after infection, cells were selected with 1 µg/ml puromycin for 7 days and the efficiency of Plexin-B2 KO was confirmed by western blot. GSC transduced with LentiCRISPRv2-sgEGFP were used as matching controls.

### Lentiviral Plexin-B2 cDNA vector**s**

Human Plexin-B2 cDNA (clone HsCD00399262, DNA Resource Core at Harvard Medical School) was modified by site-directed mutagenesis (Phusion SDM kit, Invitrogen) to generate CRISPR-resistant synonymous cDNA variant. Next, mutations in specific domains of Plexin-B2 were generated by SDM as follows: mGAP (mutation of GAP domain: R1391A, R1392G), mRBD (mutation of RBD domain: LSK 1558–1560 −> GGA), ∆VTDL (deletion of C-terminal amino acids of PDZ-binding motif), and ∆Ecto (a truncated variant that lacks extracellular domain by deletion of amino acids 30–1189). These constructs were then cloned into lentiviral vector backbone pLENTI PGK Neo DEST (Addgene 19067; deposited by Eric Campeau & Paul Kaufman) by Gateway. GSCs were infected with the Plexin-B2 cDNA lentiviruses as described above, and transduced cells were selected with 150 µg/ml G418 (Gibco).

### Cell aggregation assay in hanging drop

For cell aggregation assay, we used the hanging drop method, where 2 × 10^4^ dissociated GSC were seeded in a 10 µl droplet in neural stem cell media on an inverted lid of a 6-cm tissue culture dish. The lid was returned to a corresponding 6-cm culture dish filled with 5 ml PBS to maintain humidity during incubation. Photos of the hanging drops were taken every 24 h with a stereomicroscope. The number and size of the spheres in each drop were measured using ImageJ. Three drops/cell line per experiment were quantified, and three independent experiments were performed.

### Differential cell aggregation assay

To reveal differential adhesion between cell populations, we used the hanging drop aggregation assay with two cell populations separately labeled with green or red fluorescent dyes^36^. For cell labeling, dissociated cells were incubated in 5 µM CellTracker dye for 30 min, suing CMFDA (Invitrogen C7025) for green and CMTPX (Invitrogen C34552) for red labeling. In total, 1 × 10^4^ green cells and 1 × 10^4^ red cells were mixed evenly before seeding in each droplet. Live-cell images were taken after 24 h of incubation with an inverted Nikon fluorescent microscope.

### Cell dispersion assay

GSC cells were seeded in ultra-low-attachment U-bottom 96-well plates (Corning 7007) at a density of 2 × 10^3^ cells per well. After 5 days, single gliomaspheres formed in each U-well. Spheres were transferred to glass chamber slides (Falcon 354118) coated with 10 µg/ml laminin (Invitrogen), with 5–8 spheres per chamber, and cultured for 2–4 h in cell culture incubator, then fixed with 3.7% formaldehyde, followed by DAPI (Invitrogen) staining. Photos that focus on the bottom level of each chamber to capture migrating GSC were taken with Zeiss AXIO Imager.A2 fluorescence microscope. All cells migrating away from each sphere were counted, identified as DAPI nuclei detached from the main sphere. Cells from up to eight spheres/cell lines in each experiment were quantified, and experiments were conducted in three independent replicates.

### Limiting dilution gliomasphere assay

The sphere-forming potential of GSC was analyzed by extreme limiting dilution analysis (ELDA)^62^. Dissociated GSC was seeded in individual wells of a 96-well low-attachment plate by FACS as 1, 5, 10, or 50 cells/well. After 10 days of culture, wells containing at least one sphere were scored as positive. Quantitative analysis of sphere formation frequency was performed at the ELDA website (http://bioinf.wehi.edu.au/software/elda).

### Scratch assay

Cells were seeded in six-well plates at a density of 1 × 10^6^ cells per well. After one day culture, a scratch was made through the center of the dish with 200-µl size micropipette tips (*t* = 0 h), and would closure speed was followed by micrographs of identical areas at *t* = 24 h and 48 h after scratch.

### Durotaxis stripe assay

Polyethylene glycol diacrylate (Laysan Bio, 3.4 kDa) was mixed at 10% w/v with photo-initiator LAP (16 mM; Tocris) and fibronectin (100 μg/ml; Corning). The precursor solution was passed through a 0.22-μm syringe filter. The sterile solution was then pipetted into a 0.5-mm thick mold and exposed to collimated UV light (365 nm, 10 mW/cm^2^) for 30 s. A striped photomask was placed on top and gel was exposed to the second round of UV light for one minute. Biopsy punches were used to cut hydrogel in circle-shape, then the hydrogels were placed into well plates for cell seeding. SD2 control and PB2-KO cells were sparsely seeded on the gel, then cultured in NeuroCult proliferation medium (Stemcell Technologies) for up to 10 days.

### GSC culture in 3D fibrin gel and 3D vascular system

SD2 GSCs were labeled with mCherry expressing lentivirus (pLV-mCherry; Addgene plasmid 36084) and then cultured in 3D fibrin gel (10 mg/ml fibrinogen +3 U/ml thrombin; Sigma-Aldrich), and images were taken after 27 days of culture. Primary human brain microvascular endothelial cells (bEC; Cell Systems) expressing EGFP were first cultured on laminin-coated tissue culture flasks with endothelial cell growth medium 2 (EGM2; Promocell). For bEC spheroid formation, bEC were cultured in hanging drop condition with 0.0625% Methylcellulose (Sigma) in media. Each hanging drop containing 1000 endothelial cells was cultured for 24–36 h. Human astrocytes and brain vascular pericytes were cultured with astrocyte medium and pericyte medium (cells and media from Sciencell). To create a 3D brain microenvironment to assay GSC invasion, three cell types (astrocytes, pericytes, and bEC spheroids) were embedded in the fibrin matrix (10 mg/ml fibrinogen +3 U/ml thrombin; Sigma-Aldrich) along with sparsely distributed GSC. Seeding densities of cells were 3 million cells/ml for astrocytes and pericytes, 6 million cells/ml for bECs, and 25,000 cells/ml for GSCs. The 3D constructs with four cell types were cultured in EGM2 medium for 14 days and then imaged using fluorescence microscopy (Nikon Eclipse Ti2).

### RNA-seq of GSCs

RNA was collected from control or Plexin-B2 KO GSC (SD1–SD4) cultured on laminin-coated dishes with RNeasy (QIAGEN), and libraries for Illumina Next-Generation sequencing were prepared with NEBNext Ultra Directional RNA Library Prep Kit (NEB E7420). Sequencing was performed on HiSeq2000 and HiSeq2500 machines at Macrogen, Inc. and at New York Genome Center. The quality of sequencing reads was assessed using fastQC^63^. Reads were mapped against the human genome (hg19) and human rRNA sequences using ContextMap version 2.7.9^64^ (using BWA^65^ as a short-read aligner and default parameters). The number of reading fragments per gene was determined from the mapped RNA-seq reads using featureCounts (strand-specific for stranded libraries, non-strand-specific otherwise)^66^. For SD2 and SD3, independent triplicate libraries were prepared for sequencing. For SD1 and SD4, libraries from three clonal lines of wild-type and PB2-KO genotype were prepared for each line.

### Pathway analysis of gene expression changes

Differential gene expression analysis was performed using DESeq2^67^. For each cell line, DEGs were called at FDR < 20%. The union of all the DEGs identified in each cell line after removing discordant DEGs between cell lines was compiled as a final set of 1289 DEG (448 downregulated, 841 upregulated), which was used for functional annotation using Gene Ontology (GO)^68^, Molecular Signatures Database (MSigDB)^69,70^ and WikiPathways^71^. Data were accessed in March 2018.

For analysis of genes correlated with TCGA GBM data, level 3 TCGA GBM RNA-seqV2 (normalized) and Affymetrix mRNA data were downloaded from the Broad GDAC Firehose. For both datasets, non-primary tumors were removed. For samples present in both RNA-seq and Affymetrix datasets, only RNA-seq data were used. RNA-seq data were log2(x + 1) transformed, quantile-normalized and corrected for age, gender, and batch. Affymetrix microarray data were quantile-normalized, corrected for age, gender, and batch. For both datasets, outlier samples were detected by hierarchical clustering and removed, yielding final datasets of *n* = 150 (RNA-seq) and *n* = 378 (Affymetrix), and low-variance genes were removed. All gene symbols were updated using the MyGene.info R package^72^. Where multiple gene IDs mapped to a single gene symbol, only the highest variance entry was retained. Within each dataset, Spearman correlation of *PLXNB2* with all other genes was computed, and correlations with Benjamini–Hochberg-adjusted *P* value < 0.10 were called significant. The intersection of *PLXNB2*-correlated genes in RNA-seq and Affymetrix datasets was called the consensus *PLXNB2*-correlated genes and used for functional annotation with GO^68^, MSigDB^69,70^, and WikiPathway datasets^71^. Data were accessed in March 2018. All analyses were performed in the R statistical computing environment.

Gene set enrichment analysis (GSEA) for all expressed genes, ranked by their differential expression (Plexin-B2 KO vs. control), was performed against h.all hallmark gene sets, c2.all curated gene sets, and a matrix of gene sets associated with the matrisome gene set (http://software.broadinstitute.org/gsea/index.jsp; accessed 04/2018^69^).

### Rap1/2 GTP-loading assay

To measure GTP loading of active Rap1 and Rap2 in GSC, dissociated cells were seeded (2 × 10^5^ cells per well) in ultra-low-attachment 24-well plates. Cells formed aggregates over 48 h on a horizontal shaker inside an incubator. Aggregates were stimulated before lysis with 100 µM forskolin (Cayman Chemical) for 30 min at 37 °C. For each condition, cell aggregates from 12 wells were pooled for protein lysis using the Active Rap1 Detection Kit (Cell Signaling Technologies #8818) based on the pulldown of Rap1/2 GTP with the Rap-GTP binding domain of RalGDS. Detection of Rap proteins on Western blot membranes was performed with anti-Rap1 (host: rabbit; CST #8825) and anti-Rap2 (host: mouse; BD Biosciences #610215).

### Time-lapse live-cell imaging visualized by CellMask and Hoechst staining

SD2 and SD3 GSC were incubated for 10 min with green fluorescent dye CellMask to label cell plasma membrane (Thermo Fisher) and Hoechst 33342 (1:10,000; BD Pharmingen) for nuclear labeling in a four-chamber glass-bottom dish (Cellvis) that was coated with laminin and imaged using Leica DMi8 for live videography of dynamic changes of plasma membranes and cellular borders for 90 minutes. Frames were taken every 3 min. Video frames were analyzed with ImageJ, and composite videos were generated with Filmora9 (Wondershare).

### Analysis of pMLC2, SPY-actin, and YAP signals

Relative pMLC2 IF intensity was measured with the corrected total cell fluorescence quantification (CTCF) method with ImageJ. Cells were manually selected on micrographs, and CTCF for pMLC2 was calculated for each cell as (integrated density of pMLC2 immunosignal) − (area of cell × mean fluorescence background reading). A similar procedure was used for SPY-actin signals of live F-actin imaging. For this, we stained cells with SPY-actin 555 for one hour (Cytoskeleton) before media was changed and cells were imaged with a confocal Zeiss LSM 780 inverted microscope.

For analysis of YAP localization, cells were seeded at about 50% density into laminin-coated 8-well chamber slides (Falcon), and fixed 24 h later with 3.7% formaldehyde in PBS. Cells were stained by IF for YAP/TAZ (see section Primary Antibodies). Quantification for YAP signal was performed by manually scoring cells in selected regions of micrograph images as either nuclear signal > cytoplasmic (N > C), N < C, or N = C, respectively.

### Statistics and reproducibility

Statistical analysis was performed with Graphpad Prism v8.0 software. Analyses of experiments with two groups were performed with an unpaired *t* test, and analyses of multiple groups were performed with one-way ANOVA, followed by Dunnett’s multiple comparison test. Sample sizes are specified in figure legends. Error bars show the standard error of the mean.

No specific inclusion and exclusion criteria were used for any data or subjects. No methods were used to determine whether the data met assumptions of the statistical approach. For all analyses, *P < 0.05; **P < 0.01; ***P < 0.001; ns, not significant.

### Reporting summary

Further information on research design is available in the Nature Research Reporting Summary linked to this article.

## Supplementary information

Supplementary Information

Description of Additional Supplementary Files

Supplementary Movie 1

Supplementary Movie 2

Supplementary Movie 3

Supplementary Movie 4

Supplementary Data 1

Reporting Summary

## Data Availability

RNA-seq data of GSC lines have been deposited at the NCBI Gene Expression Omnibus (GEO) database under accession number GSE126658. Plasmids have been deposited at Addgene.org. Source data underlying the graphs is provided in Supplementary Data 1. Full western blot images are shown in Supplementary Figs. 10 and 11. All other data that support the findings of this study are available from the corresponding author R.H.F. upon reasonable request.
