# Supplementary material for: Plexin-B2 facilitates glioblastoma infiltration by modulating cell biomechanics
Source: Commun Biol. 2021 Jan 29;4:145. doi: 10.1038/s42003-021-01667-4 (PMC7846610; doi:10.1038/s42003-021-01667-4)
Supplement: Supplementary file 1 — Supplementary Information [file 42003_2021_1667_MOESM1_ESM.pdf]

Huang et al.

Plexin-B2 facilitates glioblastoma infiltration by modulating cell biomechanics

SUPPLEMENTARY FIGURES

a

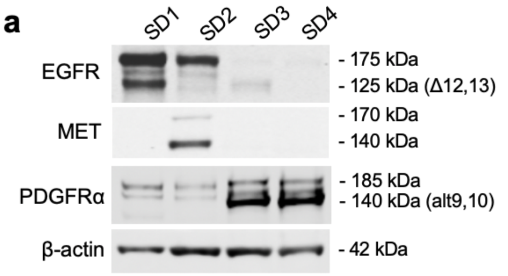

b Molecular subtype of patient-derived GSC lines

	gliovis.bioinfo.cnio.es			SubtypeMe		PCR	
	svm_call	knn_call	gsea_call	equal.call	majority.call	IDH1 (R100/109/132)	IDH2 (R140/149/172)
SD1	Classical	Classical	Classical	TRUE	**Classical**	wild-type	wild-type
SD2	Mesenchymal	Classical	Mesenchymal	FALSE	**Mesenchymal**	wild-type	wild-type
SD3	Proneural	Proneural	Classical	FALSE	**Proneural**	wild-type	wild-type
SD4	Classical	Proneural	Proneural	FALSE	**Proneural**	wild-type	wild-type

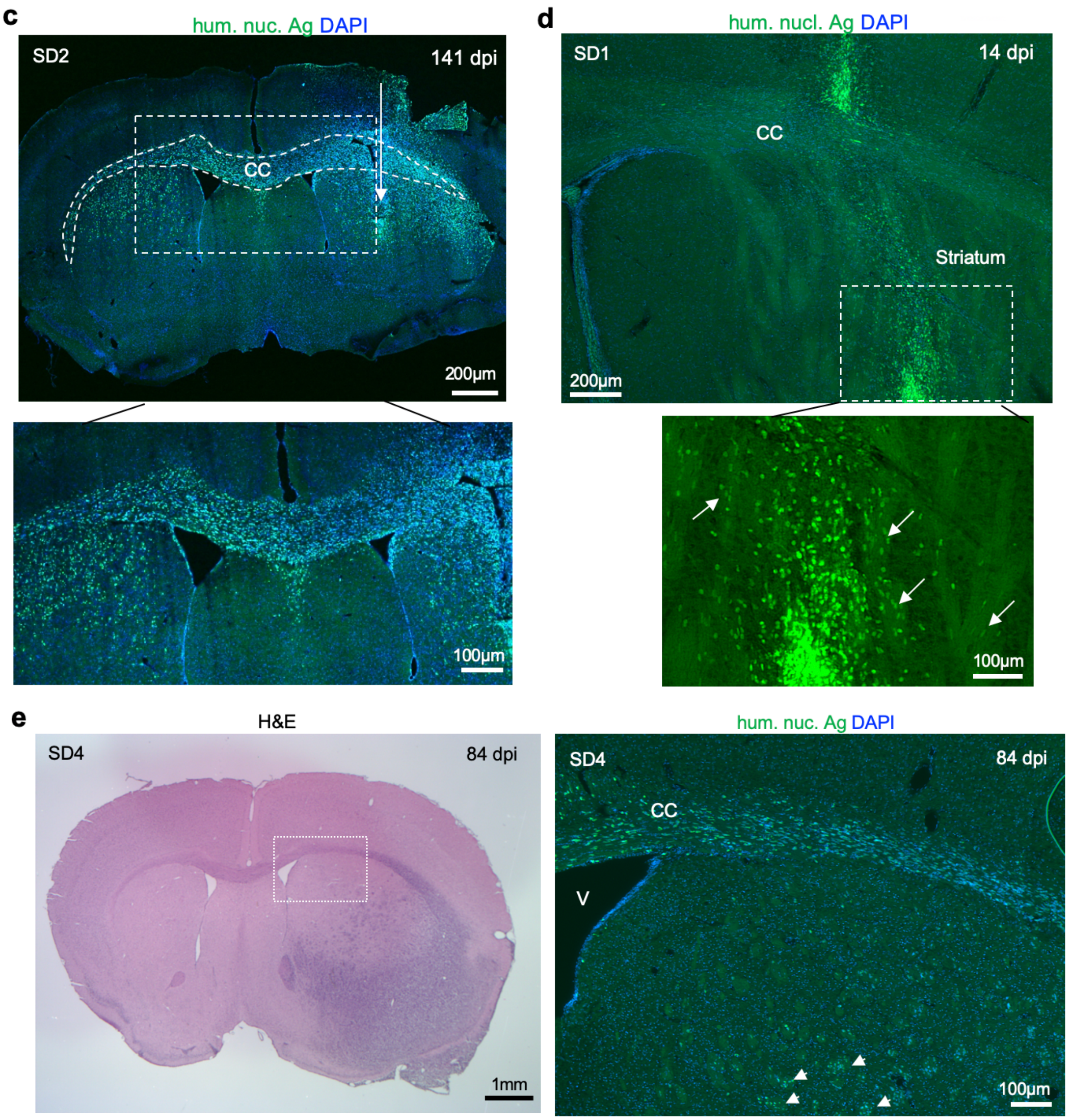

Supplementary Fig. 1 GBM stem cell lines are molecularly diverse and display diffuse infiltration in intracranial transplants.

a WB shows distinct expression of receptor tyrosine kinases (RTKs) in GSC lines. SD1 cells express full-length and a truncated EGFR (lacking exons 12 and 13 (Δ12,13); predicted by RNA-seq data). SD2 express EGFR and MET, while SD3 and SD4 highly express full-length and truncated PDGFRα (by alternative splicing between exons 9 and 10 (alt9,10); RNA-seq predicted).

b GBM subtype analysis of GSCs based on RNA-seq data with GlioVis platform (gliovis.bioinfo.cnio.es). The majority calls of three algorithms classify SD1 as classical, SD2 mesenchymal, and SD3 and SD4 proneural molecular subtypes. Right side of

table indicates IDH1 and IDH2 wild-type genotypes as determined by Sanger sequencing.

c IF images of coronal section of brain transplanted with SD2 GSCs into right striatum (arrow indicates injection track) stained for human nuclear antigen. At 141 dpi, GBM cells had widely disseminated in the striatum and also deep into contralateral striatum along the corpus callosum (CC, outlined by dashed lines). DAPI for nuclear counterstaining.

d IF image of coronal section of the brain transplanted with SD1 GSCs at 14 dpi. Note early dissociation of tumor cells from tumor bulk and invasion into striatum and CC. Bottom: Enlarged image of boxed area, highlighting invasion of tumor cells along striatal axon fiber bundles (arrows).

e Left: Coronal section of the brain transplanted with SD4 GSCs at 84 dpi, stained with hematoxylin/eosin (H&E), showing tumor spreading into CC and striatum. Right, IF image of adjacent section (corresponding to boxed area) shows aggressive invasion of tumor cells inside striatal fiber bundles (arrows) and along corpus callosum (CC).

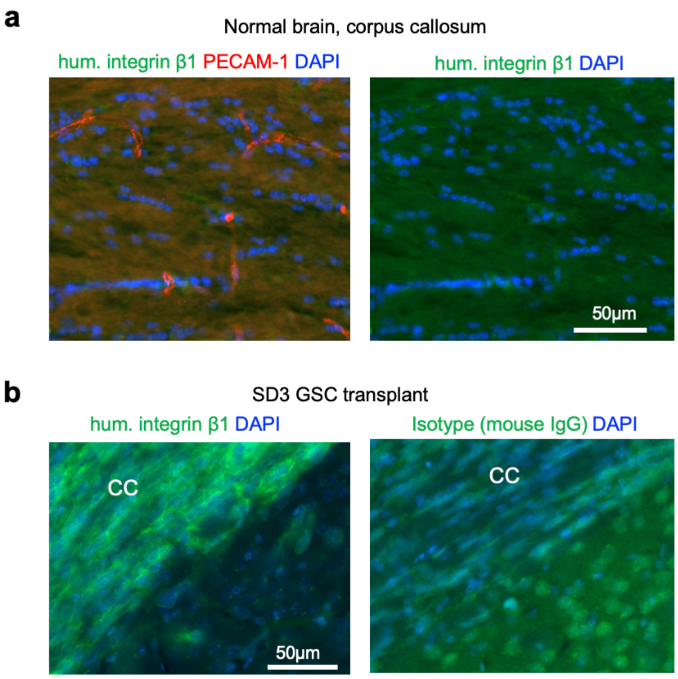

Supplementary Fig. 2 Control immunofluorescence staining for anti-human integrin β1.

a IF images of corpus callosum area in normal adult mouse brain show positive immunosignals for vasculature (PECAM-1^{+}), but absence of specific signals with human-specific anti-integrin β1 antibody (clone TS2/16).

b Left, IF staining of SD3 GSC transplant with human-specific anti-integrin β1 antibody (TS2/16) detects abundant infiltrating tumor cells in the corpus callosum (CC). Right, isotype control IF staining with purified mouse IgG serum (2 μg/ml) did not generate specific IF signals.

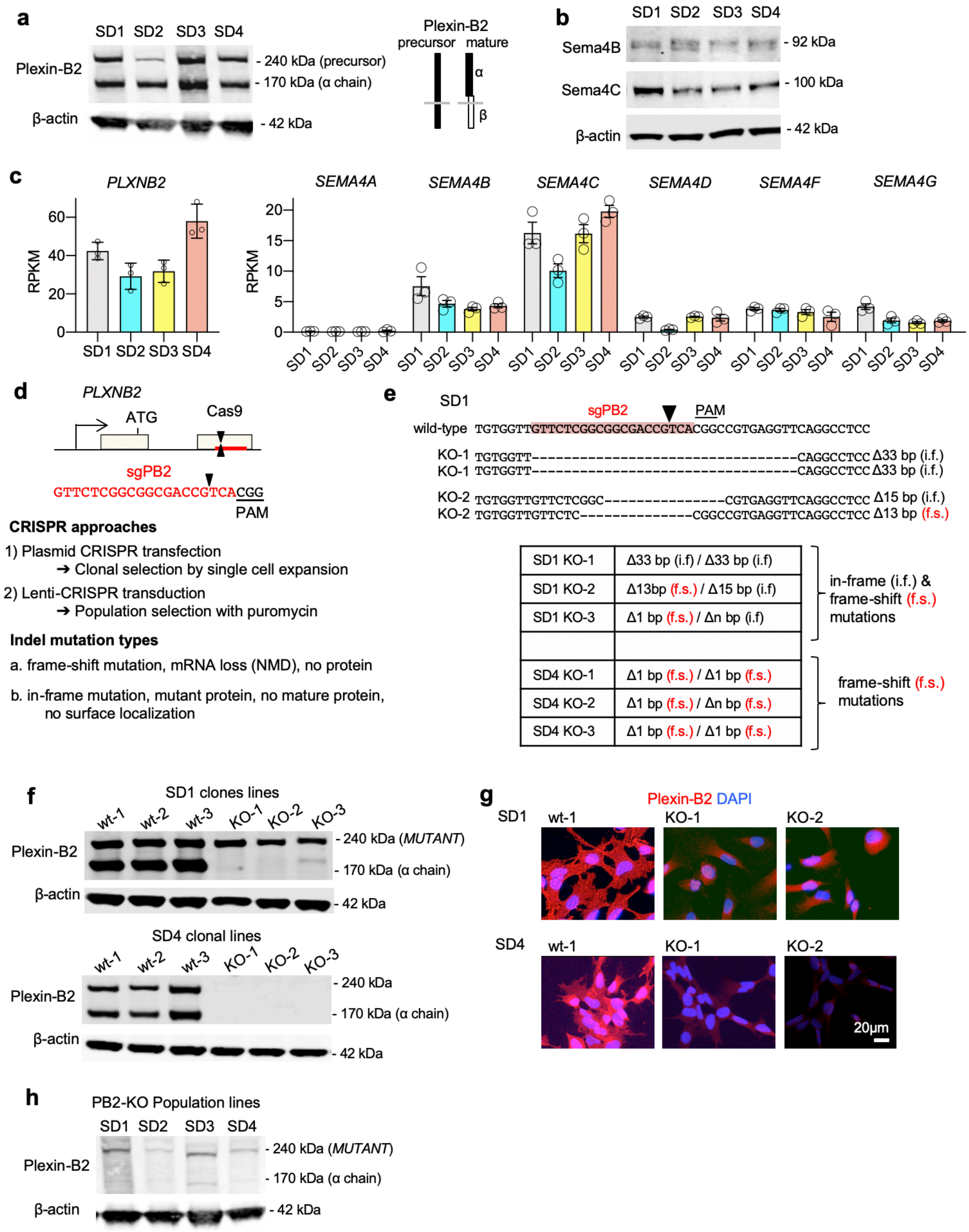

Supplementary Fig. 3 Plexin-B2 and Sema4 expression in GSC / CRISPR/Cas9 knockout of *PLXNB2*

a WB of SD1-SD4 GSC lines shows robust Plexin-B2 protein expression in all lines. WB image of SD2 and SD3 GSCs of Fig. 2a is repeated here for comparison.

b WBs of GSC lines SD1-SD4 show expression of Sema4B and Sema4C, which are potential ligands for Plexin-B2.

c Gene expression levels of Plexin-B2 and Sema4 genes measured by RNA-seq as RPKM (reads-per-kb-per-million bases). Sema4C has the highest mRNA levels in GSC lines, followed by Sema4B, 4D, 4F, and 4G are expressed at lower levels. Also note that SD2 expresses lower level of Sema4C and 4B than SD3.

d Top: CRISPR/Cas9 strategy to target the second coding exon of *PLXNB2* by sgRNA for cut (black triangle), which will create indel (insertion/deletion) mutations. PAM, protospacer adjacent motif. Bottom: Summary of plasmid and lentiviral CRISPR/Cas9 approaches and of possible indel mutation types.

e *PLXNB2* alleles were sequenced in two clonal CRISPR/Cas9 KO lines of SD1. Line KO-1 carries bi-allelic in-frame deletion of 33 bp and line KO-2 carries one in-frame deletion of 15 bp and one frame-shift deletion of 13 bp.

f WB shows absence of mature Plexin-B2 **α** chain (170 kDa) in clonal PB2-KO lines of SD1 and SD4. A mutant precursor form of Plexin-B2 (240 kDa) is detectable in some KO lines due to in-frame mutations.

g IF images demonstrate loss of surface Plexin-B2 expression in KO clonal lines of SD1 and SD4 GSCs.

h WB of SD1-SD4 population lines with Plexin-B2 KO. These PB2-KO lines are polyclonal with in-frame and frame-shift mutations. In-frame mutations create a detectable mutant precursor proteins (*MUTANT*) at ~240 kDa that are not processed to mature α/β chain heterodimer. Note absence of mature Plexin-B2 **α** chain at 170 kDa.

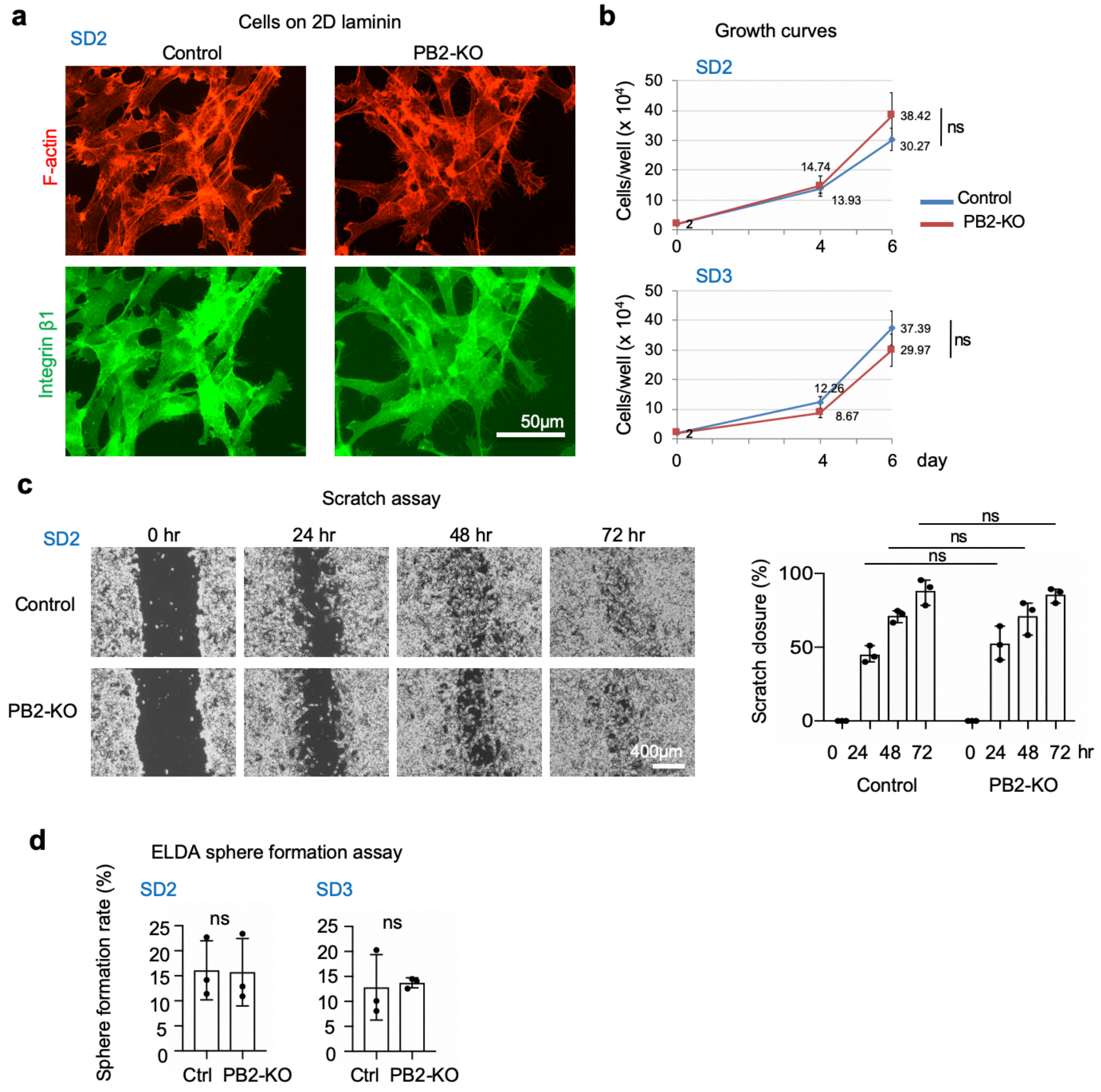

Supplementary Fig. 4 Plexin-B2 deletion does not significantly alter cell behaviors in 2D culture assays.

a Control and PB2-KO SD2 GSCs cultured on 2D laminin-coated dishes displayed no apparent differences in cell morphology revealed by staining for filamentous actin (F-actin; phalloidin staining) or for human integrin β1.

b Growth curves of SD2 and SD3 GSCs (control vs. PB2-KO), cultured on 2D laminin-coated dishes, show no significant effects of Plexin-B2 deletion on proliferation rate. Two-way ANOVA test; ns, not statistically significant.

c Photos of scratch wound assays show similar wound closure speed for control and Plexin-B2 KO SD2 GSCs. n = 3 independent experiments, 12 fields/group/replicate scored; p = 0.26, 0.98, and 0.74 for 24, 48, and 72 hr, respectively; paired Student's t-test.

d Limiting dilution sphere formation assay, showing that Plexin-B2 deletion did not affect stem cell frequency in either SD2 or SD3 GSCs, calculated using ELDA software. n = 3 independent replicates; p = 0.59 for SD2 and p = 0.85 for SD3; paired Student's t-test.

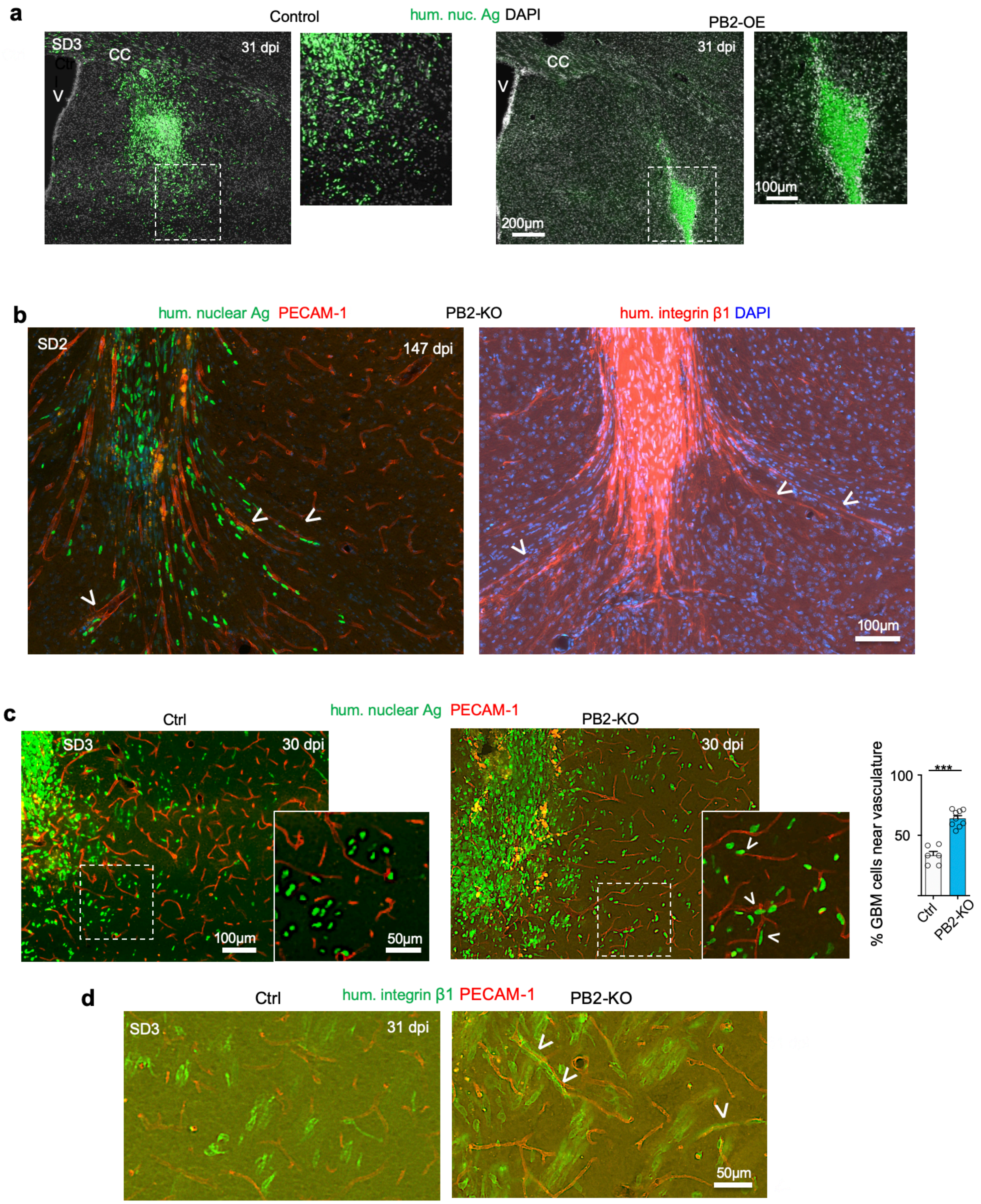

Supplementary Fig. 5 Plexin-B2 overexpression limits GBM spread; Plexin-B2 ablation shifts preferred migration route to perivascular route.

a Representative IF images of coronal sections of brains transplanted with SD3 GSCs at 31 dpi show that PB2 OE impeded GBM invasion. Enlarged images of boxed area are shown on the right, highlighting diffuse spread of tumor cells (stained for human nuclear antigen) in control GBM, but accumulation of tumor cells near engraftment site in PB2 OE transplant.

b IF images of consecutive sections of striatum highlight cell morphology and orientation of invading tumor cells (outlined by human integrin β1) in close alignment with the axes of microvasculature (PECAM-1) (arrowheads).

c Representative IF images of coronal sections of brains with SD3 transplants show shift of preferred migratory path from axon fiber tracts in control GBM to along small vessels in PB2 KO GBM (arrowheads). Enlarged images of boxed area are shown on the right. Right: Quantification of invading tumor cells in association with vasculature. n = 6-9 areas from 3 independent transplants; unpaired t-test; *** $p<0.001$.

d IF images of coronal sections of striatum highlight tumor cell morphology and orientation as outlined by human integrin β1, and their spatial relationship with microvasculature (PECAM-1). In control SD3 transplant, invading GBM cells adhere to striatal fiber bundles surrounded by blood vessels at the periphery, with cell orientation in cross section from the coronal view. PB2 KO resulted in increased numbers of GBM cells that changed their orientation to align with vessel axes (arrowheads).

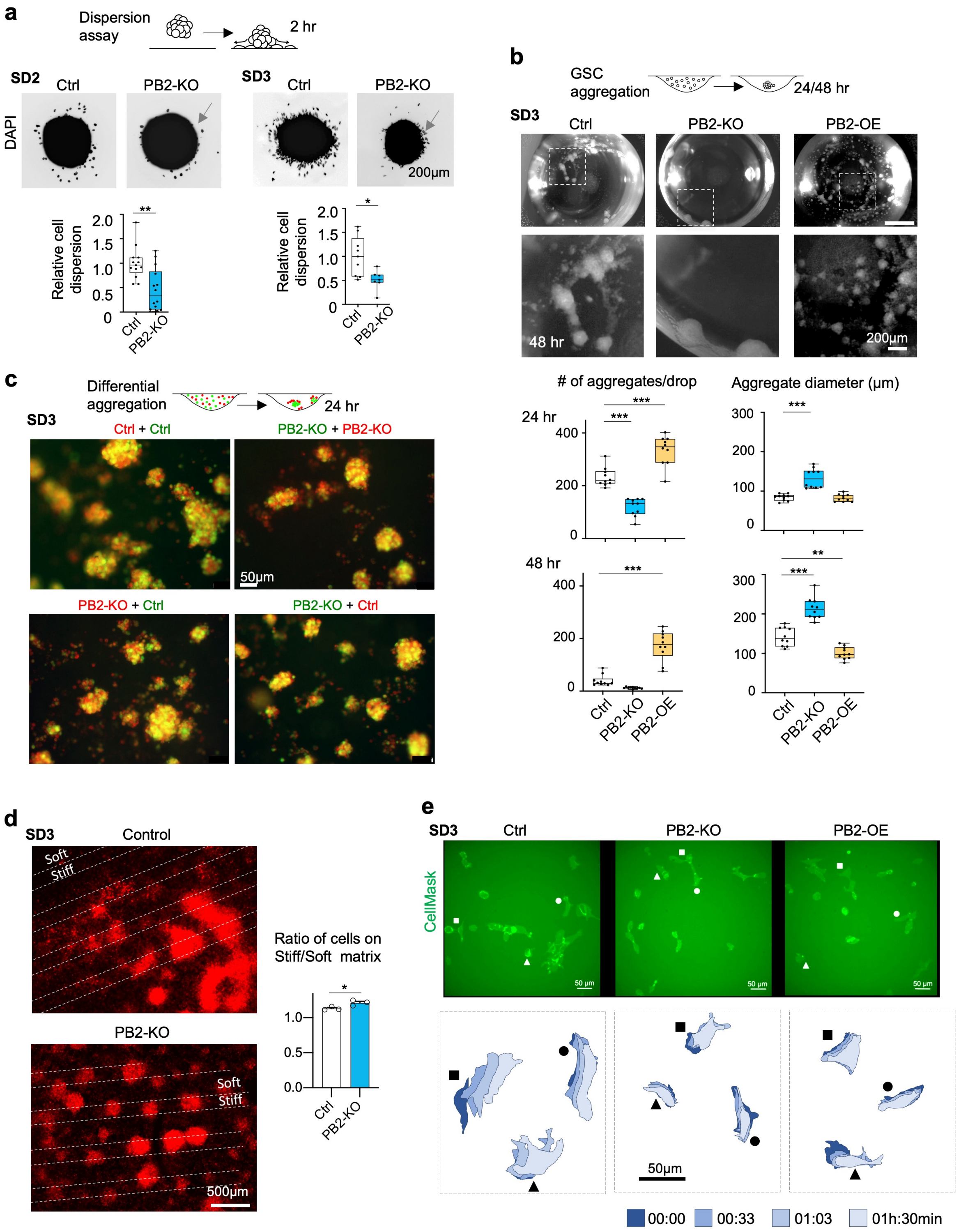

Supplementary Fig. 6 Plexin-B2 reduces intercellular adhesiveness of GSC and alters durotaxis behavior.

a Cell dispersion assay. GSC aggregates were placed on laminin, and cells that detached from aggregate and dispersed on culture surface are quantified after 2 hours. Representative images and quantifications of cell dispersion from SD2 and SD3 wild-type and PB2 KO aggregates after 2 hours are shown.

b Hanging drop aggregation assay. Representative photos of aggerates of SD3 wild-type, PB2-KO, and PB2-OE GSCs at 48 hours after seeding and quantification of numbers and sizes of aggregates in hanging drops at 24 or 48 hours are shown. n = 5-11 hanging drops per group; *p<0.05, ***p<0.001; one-way ANOVA with Dunnett's multiple comparison test of each group against control.

c Differential aggregation assay. Cells were separately labeled with green or red CellTracker dyes and mixed 1:1 before seeding in hanging drops. Example images of aggregated spheres of SD3 GSC of different genotypes are shown. No differential cell distributions were detectable. This assay may not be not well-suited for SD3 GSCs (in contrast to SD2 GSCs, see Fig. 4), due to faster aggregation rates and large spheres of SD3 GSCs.

d Durotaxis stripe assay. SD3 GSCs expressing lenti-mCherry were cultured for 15 days on alternating stripes of soft (~25 kPa) and stiff (~30 kPa) PEG substrate. PB2-KO cells revealed a preference for aggregation on stiff stripes. For quantification, we measured mCherry fluorescence signals from soft vs. stiff stripes. n = 3 independent experiments per group. *p=0.04, unpaired t-test.

e Live-cell imaging. Top, still frames from time lapse live-imaging of SD3 GSCs labeled with CellMask membrane dye. Bottom, overlapping cell contour plots of three randomly selected cells in each group at 30 min-intervals over 90 mins. Note more dynamic movement of control GSCs, but more static locomotion for both PB2 KO and OE conditions.

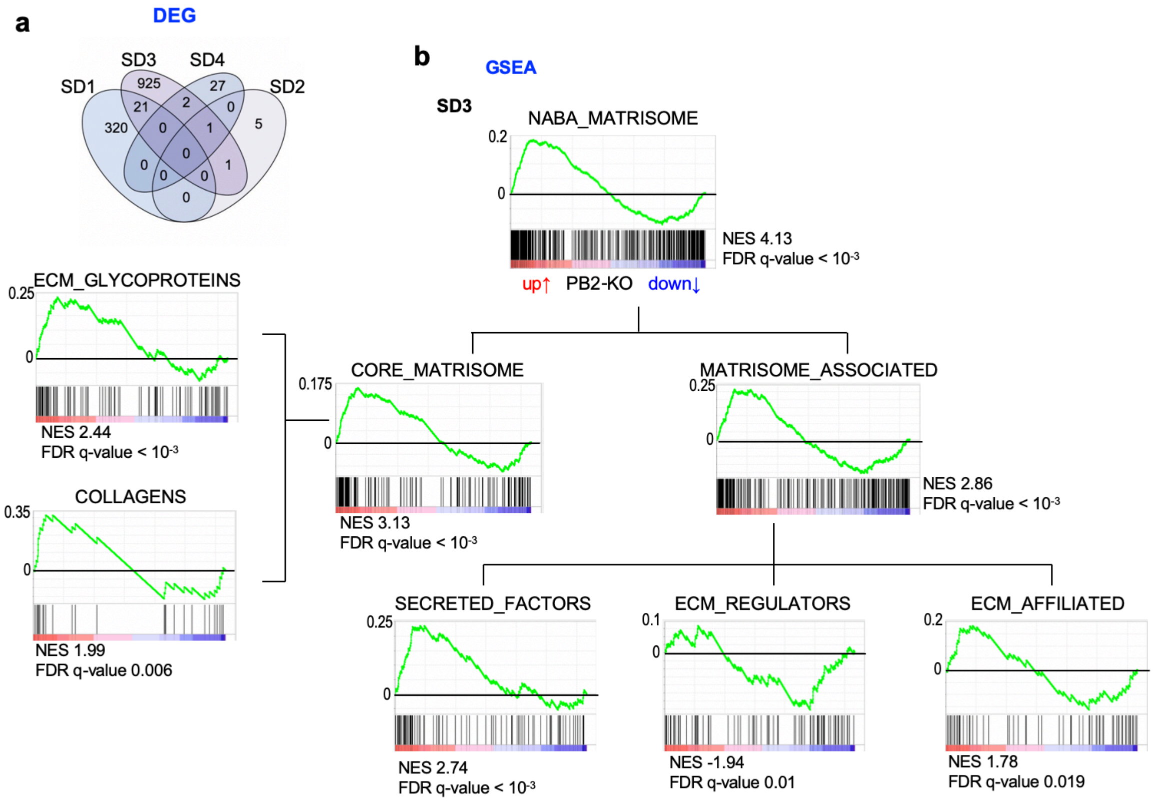

Supplementary Fig. 7 Gene set enrichment analysis of matrisome subsets for SD3 GSC.

a Differentially expressed genes (DEGs) of PB2-KO GSCs relative to controls for four different patient-derived lines (FDR<20%). Intersection revealed few shared DEGs between different GSC lines upon deletion of Plexin-B2.

b Gene set enrichment analysis (GSEA) of SD3 GSCs (PB2-KO relative to control) show enrichment of all Matrisome subsets (Naba et al., 2012). Of note, the gene set ECM_Affiliated also contains semaphorins and plexins, including Plexin-B2.

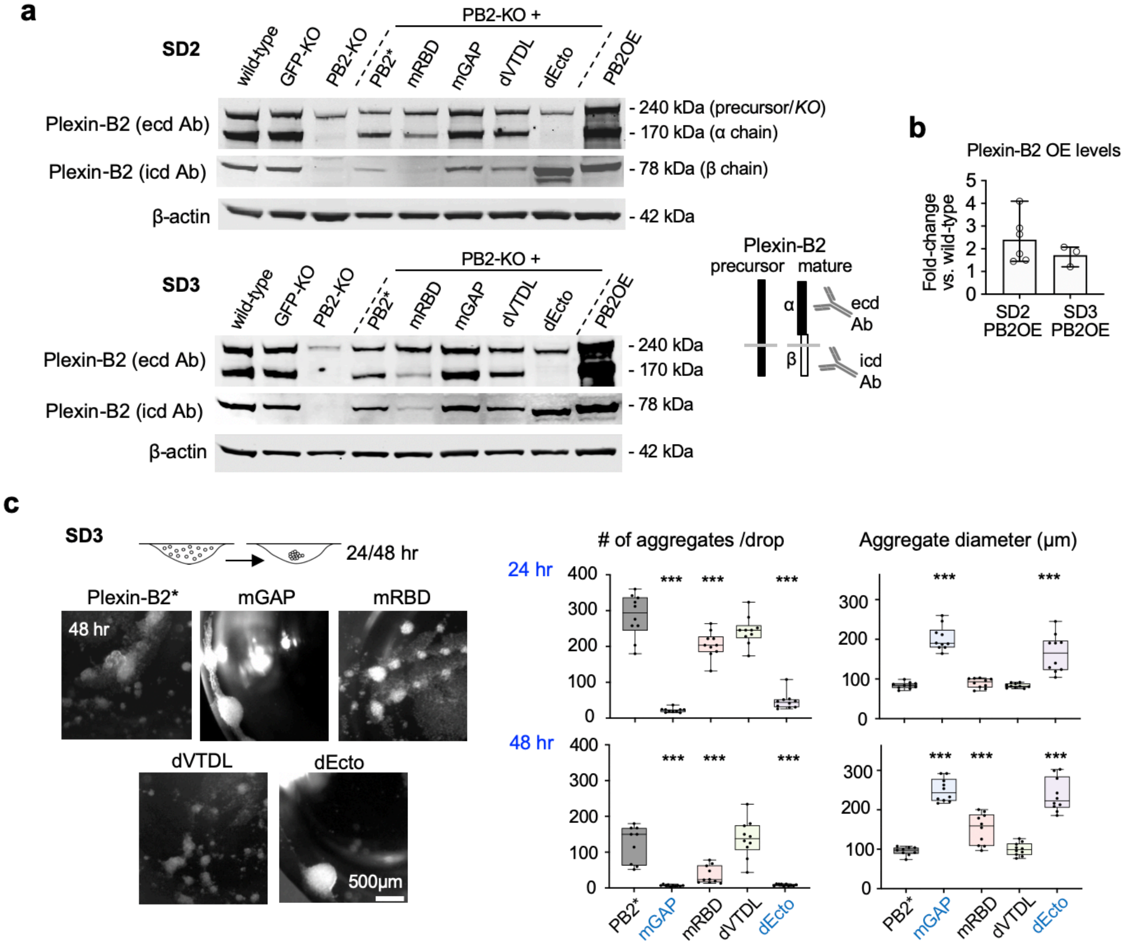

Supplementary Fig. 8 Expression of Plexin-B2 signaling mutants and effect on aggregation of SD3 GSCs.

a WB of SD2 and SD3 GSC lines with antibodies against Plexin-B2 precursor/ectodomain (ectodomain antibody, ecd Ab) or intracellular domain (icd Ab). Note that WBs show comparable expression levels of Plexin-B2 signaling mutants that were introduced into PB2-KO GSCs (SD2 or SD3) with lentiviral vectors.

b Quantification of Plexin-B2 overexpression (OE) in SD2 and SD3 PB2-OE GSC lines (wild-type GSCs were transduced with lentivirus for Plexin-B2 expression). SD2- and SD3-PB2-OE cells expressed on average of 2.4- and 1.7-fold levels of Pleixn-B2 relative to that in wild-type controls, respectively. n = 6 independent measurements for SD2 OE, and n=3 for SD3 OE.

c Hanging drop cell aggregation assay for SD3 GSCs expressing Plexin-B2 signaling mutants. Images on left show representative examples of drops after 48 hours. Quantifications on the right show numbers and sizes of spheres in hanging drops at 24 and 48 hours. Mutations in Ras-GAP domain (mGAP) or deletion of extracellular domain (dEcto) failed to rescue the KO phenotype (marked as blue). n = 5-11 drops per group; one-way ANOVA with Dunnett's multiple comparison test of each group; *p<0.05, ***p<0.001.

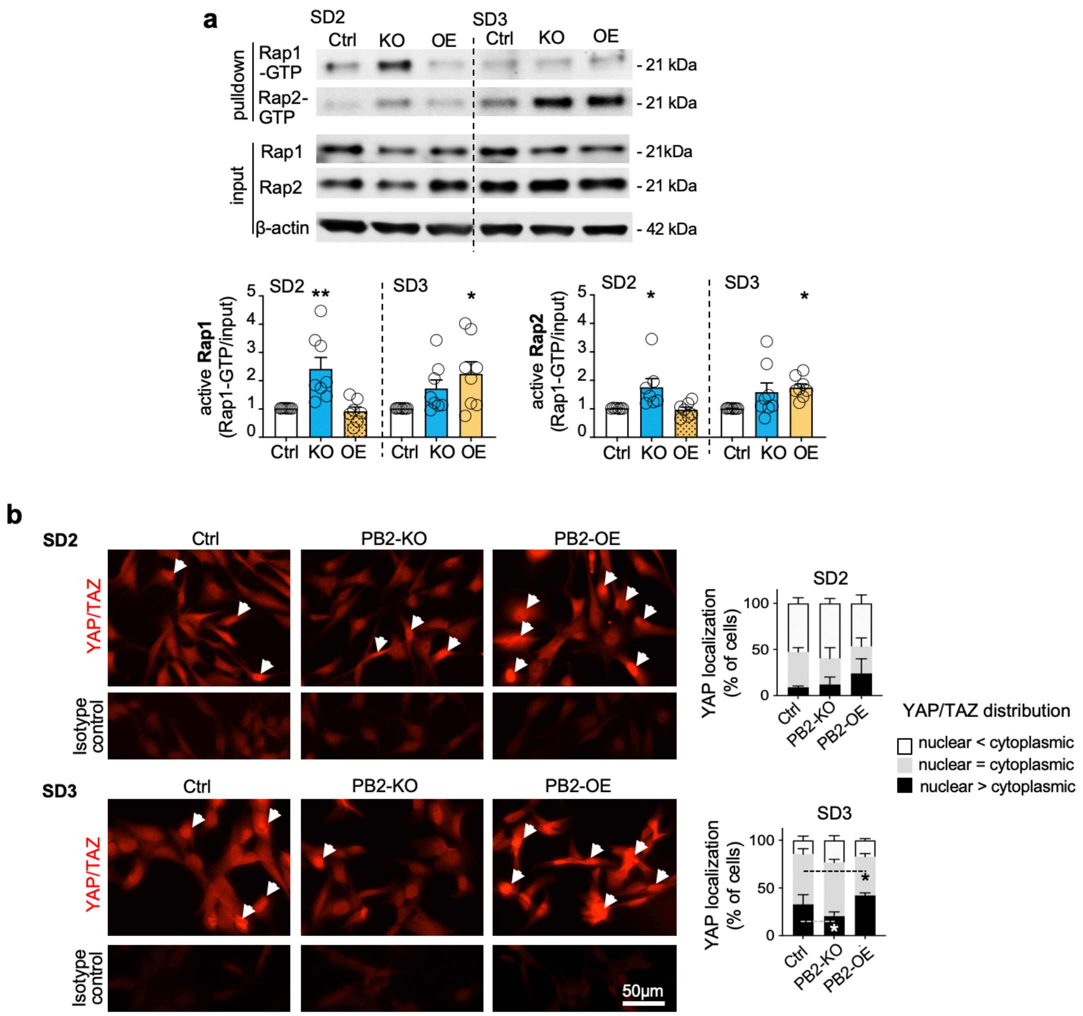

Supplementary Fig. 9 Impact of Plexin-B2 function on Rap1/2 activation and YAP nuclear localization.

a WBs show levels of total Rap1 and Rap2 (input), and active Rap1 and Rap2 (GTP-bound, pulldown) in GSCs. GSCs were grown as 3D spheres for 2 days and stimulated before lysis for 30 min with forskolin (100 µM) to upregulate Rap1/2 activity. In control conditions, Rap1 was more active than Rap2 in SD2 GSCs, while Rap2 was more active in SD3 GSCs. PB2 KO increased GTP-bound Rap1 and Rap2 in both SD2 and SD3. PB2-OE led to no significant changes of active Rap1/2 in SD2 GSCs, but surprisingly increased active Rap2 in SD3, suggesting that Plexin-B2 may not be sufficient by itself to reduce Rap1/2 activity. Of note, anti-Rap1 and anti-Rap2 antibodies detect the isoforms Rap1A/B and Rap2A/B/C, respectively, which are all expressed in the GSCs (RNA-seq data). Quantification shows levels of GTP-bound active Rap1/2 relative to input and normalized to control cells. n = 7-9 independent experiments; one-way ANOVA with Dunnett's multi-comparison test; * $p<0.05$, ** $p<0.01$.

b Immunocytochemistry images show trend of increased nuclear localization of YAP/TAZ in PB2-OE GSCs (arrows). Quantifications show percentages of cells with nucleus vs. cytoplasm distribution of YAP/TAZ. n = 3 independent experiments with >100 cells counted for each PB2 genotype condition. Comparisons between Ctrl cells and PB2 KO and OE. 2-way ANOVA with Dunnett's multiple comparison test. * $p<0.05$. Differences for SD2 GSCs were not statistically significant.

Supplementary Fig. 10

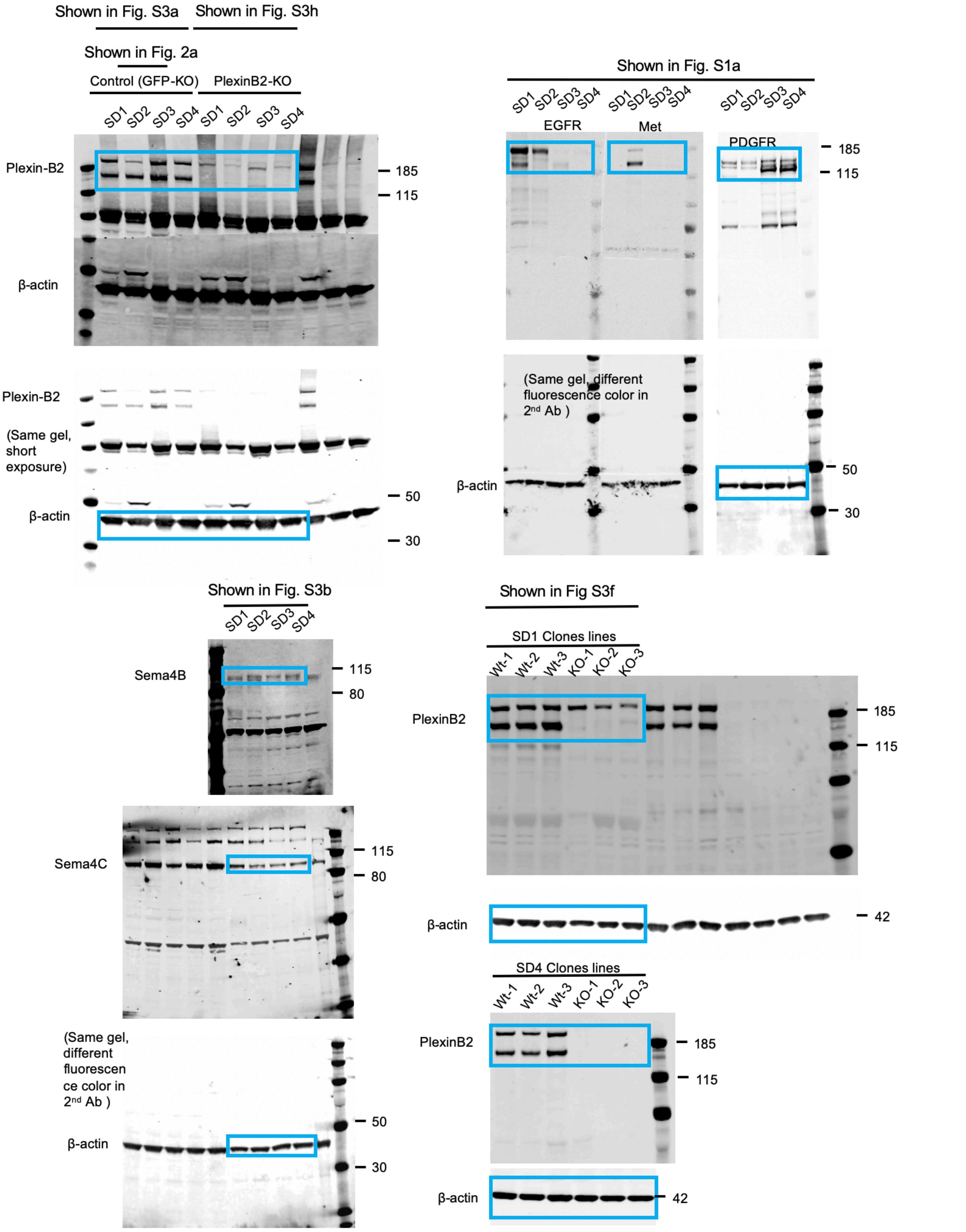

Supplementary Fig. 10 Uncropped membranes for Western blots that are shown in Fig. 2a, S1a, S3a, S3b, S3f, S3h.

Supplementary Fig. 11

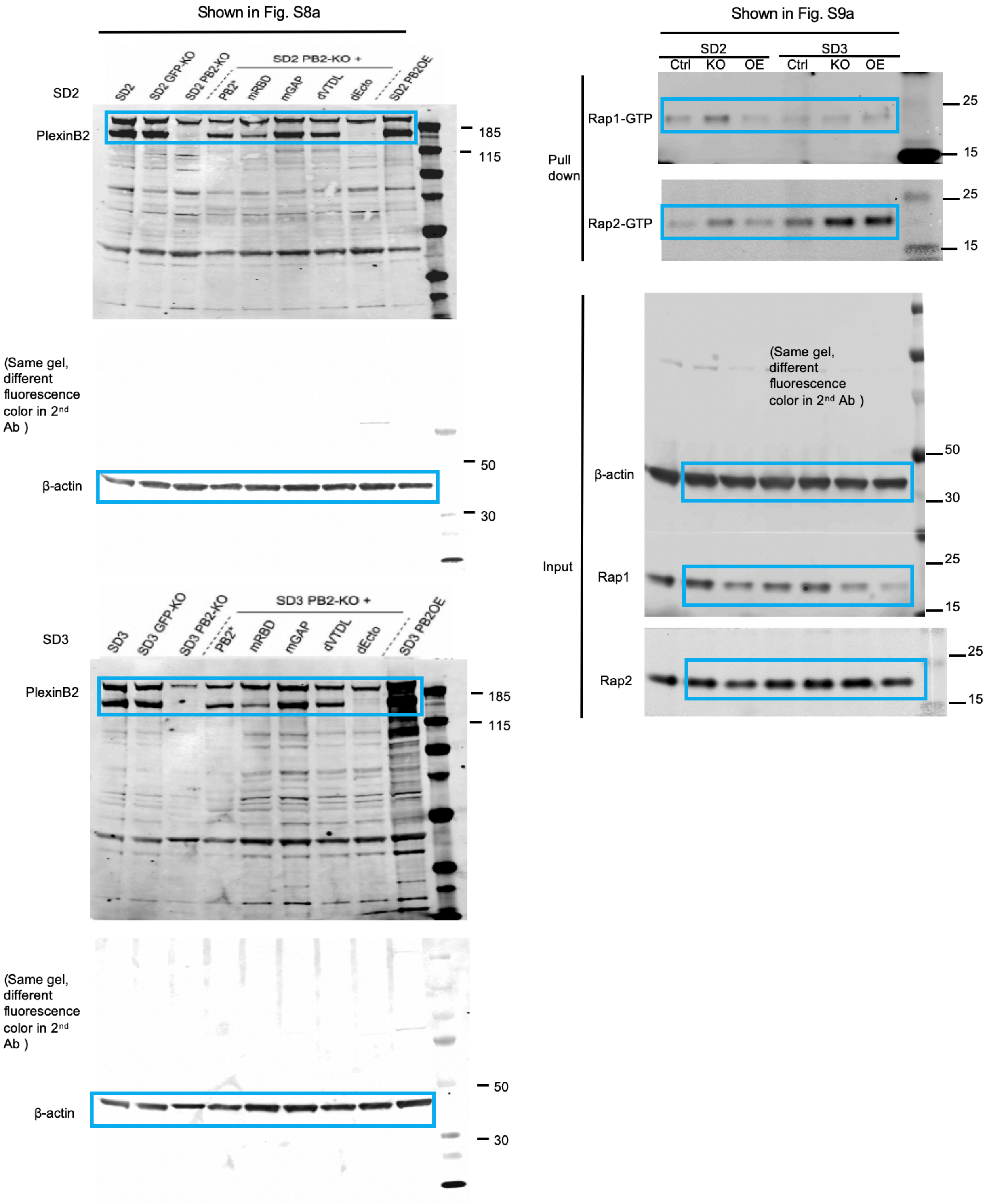

Supplementary Fig. 11 Uncropped membranes for Western blots shown in Fig. S8a, S9a.
