# Supplementary material for: Plexin-B2 facilitates glioblastoma infiltration by modulating cell biomechanics
Source: Commun Biol. 2021 Jan 29;4:145. doi: 10.1038/s42003-021-01667-4 (PMC7846610; doi:10.1038/s42003-021-01667-4)
Supplement: Supplementary file 2 — Description of Additional Supplementary Files [file 42003_2021_1667_MOESM2_ESM.pdf]

Description of Additional Supplementary Files

File name: Supplementary Video 1. Movement of SD2 GSCs with Plexin-B2 manipulation, visualized by Hoechst nuclear staining

Description: Locomotion speed of SD2 GSC is impeded by both Plexin-B2 KO and OE. Movement of selected nuclei is tracked. See Fig. 5b for still frames and quantification.

File name: Supplementary Video 2. Movement of SD3 GSCs with Plexin-B2 manipulation, visualized by Hoechst nuclear staining

Description: Locomotion speed of SD3 GSC is impeded by Plexin-B2 KO. Movement of selected nuclei is tracked. See Fig. 5b for still frames and quantification.

File name: Supplementary Video 3. Cell dynamics of SD2 GSCs, visualized by CellMask membrane staining

Description: While control GSC displayed rapid and dynamic changes of cell localization, PB2-KO and -OE cells appeared more static in locomotion. See Fig. 5c for still frames and contour tracing of cells marked by circle, square, or triangle.

File name: Supplementary Video 4. Cell dynamics of SD3 GSCs, visualized by CellMask membrane staining

Description: While control GSC displayed rapid and dynamic changes of cell localization, PB2-KO and -OE cells appeared more static in locomotion. See Supplementary Fig. 6e for still frames and contour tracing of cells marked by circle, square, or triangle.

File name: Supplementary Data 1.

Description: Source data underlying graphs shown in figures.
