# Supplementary material for: Plexin-B2 facilitates glioblastoma infiltration by modulating cell biomechanics
Source: Commun Biol. 2021 Jan 29;4:145. doi: 10.1038/s42003-021-01667-4 (PMC7846610; doi:10.1038/s42003-021-01667-4)
Supplement: Supplementary file 8 — Reporting Summary [file 42003_2021_1667_MOESM8_ESM.pdf]

nature research

Corresponding author(s): Hongyan Zou, Roland Friedel

Last updated by author(s): Dec 2, 2020

Reporting Summary

Nature Research wishes to improve the reproducibility of the work that we publish. This form provides structure for consistency and transparency in reporting. For further information on Nature Research policies, see Authors & Referees and the Editorial Policy Checklist .

Statistics

For all statistical analyses, confirm that the following items are present in the figure legend, table legend, main text, or Methods section.

n/a	Confirmed	
☐	☒	The exact sample size (n) for each experimental group/condition, given as a discrete number and unit of measurement
☐	☒	A statement on whether measurements were taken from distinct samples or whether the same sample was measured repeatedly
☐	☒	The statistical test(s) used AND whether they are one- or two-sided<br>*Only common tests should be described solely by name; describe more complex techniques in the Methods section.*
☒	☐	A description of all covariates tested
☐	☒	A description of any assumptions or corrections, such as tests of normality and adjustment for multiple comparisons
☐	☒	A full description of the statistical parameters including central tendency (e.g. means) or other basic estimates (e.g. regression coefficient) AND variation (e.g. standard deviation) or associated estimates of uncertainty (e.g. confidence intervals)
☐	☒	For null hypothesis testing, the test statistic (e.g. F, t, r) with confidence intervals, effect sizes, degrees of freedom and P value noted<br>*Give P values as exact values whenever suitable.*
☒	☐	For Bayesian analysis, information on the choice of priors and Markov chain Monte Carlo settings
☒	☐	For hierarchical and complex designs, identification of the appropriate level for tests and full reporting of outcomes
☒	☐	Estimates of effect sizes (e.g. Cohen's d, Pearson's r), indicating how they were calculated

Our web collection on statistics for biologists contains articles on many of the points above.

Software and code

Policy information about availability of computer code

Data collection	*Provide a description of all commercial, open source and custom code used to collect the data in this study, specifying the version used OR state that no software was used.*
Data analysis	Statistical analysis was performed with Graphpad Prism v8.0 software. Differential gene expression analysis was performed using DESeq2 (Love et al., 2014).

For manuscripts utilizing custom algorithms or software that are central to the research but not yet described in published literature, software must be made available to editors/reviewers. We strongly encourage code deposition in a community repository (e.g. GitHub). See the Nature Research guidelines for submitting code & software for further information.

Data

Policy information about availability of data

All manuscripts must include a data availability statement. This statement should provide the following information, where applicable:

- Accession codes, unique identifiers, or web links for publicly available datasets
- A list of figures that have associated raw data
- A description of any restrictions on data availability

RNA-Seq data files were deposited at the National Center for Biotechnology Information (NCBI) data repository GEO, accession number GSE126658.

Field-specific reporting

Please select the one below that is the best fit for your research. If you are not sure, read the appropriate sections before making your selection.

☒ Life sciences ☐ Behavioural & social sciences ☐ Ecological, evolutionary & environmental sciences

Life sciences study design

All studies must disclose on these points even when the disclosure is negative.

Sample size	A minimum of n=4 animals up to n=12 animals per cohort were used. No statistical methods were used to pre-determine sample sizes, but our sample sizes are similar to those reported in previous publications. For histological studies, a minimum of n=3 animals per cohort/ treatment/timepoint were stained. The precise numbers of animals used in each experiment are given in the figure legend. Statistical tests were then used according to experimental outline.
Data exclusions	In most experiments, we included all data. For specific experiments, we excluded certain data / timepoints / animal pairs only after determining outliers using the Grubb's Outlier Test from GraphPad Prism online portal. No exclusion criteria were pre-established.
Replication	For all studies, independent animals were used as replicates. Control conditions between different experimental setups confirmed scores. Replication studies confirmed our results in all experiments.
Randomization	Mice were randomly assigned into control or experimental groups.
Blinding	Data collection and analysis were not performed in a blinded fashion.

Reporting for specific materials, systems and methods

We require information from authors about some types of materials, experimental systems and methods used in many studies. Here, indicate whether each material, system or method listed is relevant to your study. If you are not sure if a list item applies to your research, read the appropriate section before selecting a response.

Materials & experimental systems

n/a	Involved in the study	
☐	☒	Antibodies
☐	☒	Eukaryotic cell lines
☒	☐	Palaeontology
☐	☒	Animals and other organisms
☒	☐	Human research participants
☒	☐	Clinical data

Methods

n/a	Involved in the study	
☒	☐	ChIP-seq
☒	☐	Flow cytometry
☒	☐	MRI-based neuroimaging

Antibodies

Antibodies used

anti-β-actin, Sigma A1978 (host species: mouse), dilution 1:10,000 for WB
anti-EGFR, Cell Signaling Technology 4267 (rabbit), 1:1,000 for WB
anti-human nuclear antigen (HNA), Millipore MAB1281 (mouse), 1:250 for IF
anti-integrin β1 (human specific clone TS2/16), Santa Cruz sc-53711 (mouse), 1:300 for IF
anti-laminin, Millipore MAB1905 (rat), 1:200 for IF
anti-MBP, CST 78896 (rabbit), 1:100 for IF
anti-MET, Cell Signaling Technology 8198 (rabbit), 1:1,000 for WB
anti-pMLC 2 (Ser19), Cell Signaling Technology 3671 (rabbit), 1:100 for IF
anti-PDGFRα, Cell Signaling Technology 3174 (rabbit), 1:1,000 for WB
anti-PECAM-1/CD31, BD Biosciences 553370 (rat), 1:300 for IF
anti-Plexin-B1, R&D systems AF3749 (goat), 1:400 for WB
anti-Plexin-B2, extracellular domain, R&D systems AF5329 (sheep), 1:400 for WB, 1:100 for IF
anti-Plexin-B2, intracellular domain, Abcam ab193355 (rabbit), 1:1000 for WB
anti-Plexin-B3, R&D systems AF4958 (sheep), 1:400 for WB
anti-Sema4B, Proteintech 16934-1-AP (rabbit), 1:400 for WB
anti-Sema4C, LSBio C150056 (sheep), 1:400 for WB
anti-YAP/TAZ, Santa Cruz sc101199 (mouse), 1:100 for IF

Validation

No customized antibodies were used. The anti-Plexin-B2 antibody was validated in this paper using Plexin-B2 knockoutconditions. Validation data about the other antibodies purchased from commercial vendors are available on the manufactures' website (refer to the antibody catalogue information to locate the online link).

Eukaryotic cell lines

Policy information about cell lines

Cell line source(s)	De-identified human GBM stem cell (GSC) SD1-SD4 had been established from resected tumor tissue of GBM patients at University of California, San Diego by Dr. Kesari and Dr. Pingle (Le et al., 2015).
Authentication	Cell lines are characterized and authenticated by RNA-seq, qPCR, and Western blot for expression of marker genes PDGFRA, EGFR, and MET.
Mycoplasma contamination	Not tested.
Commonly misidentified lines (See ICLAC register)	*Name any commonly misidentified cell lines used in the study and provide a rationale for their use.*

Animals and other organisms

Policy information about studies involving animals; ARRIVE guidelines recommended for reporting animal research

Laboratory animals	Adult immunocompromised male ICR SCID mice (IcrTac:ICR-Prkdcscid) used for intracranial transplantation were purchased from Taconic Biosciences.
Wild animals	The study did not involve wild animals.
Field-collected samples	The study did not involve field-collected samples.
Ethics oversight	All animal procedures complied with ethical regulations for animal testing and research and were performed according to protocols approved by the Institutional Animal Care and Use Committee (IACUC) at Icahn School of Medicine at Mount Sinai. Animals were housed in groups of 5 in pathogen free barrier facility, in corn bedding lined cages, with pellet chow and water bottles.

Note that full information on the approval of the study protocol must also be provided in the manuscript.
